# 3DMOUSEneST: a volumetric label-free imaging method evaluating embryo–uterine interaction and decidualization efficacy

**DOI:** 10.1242/dev.202938

**Published:** 2024-08-29

**Authors:** Audrey Savolainen, Emmi Kapiainen, Veli-Pekka Ronkainen, Valerio Izzi, Martin M. Matzuk, Diana Monsivais, Renata Prunskaite-Hyyryläinen

**Affiliations:** ^1^ Faculty of Biochemistry and Molecular Medicine, University of Oulu, 90220 Oulu, Finland; ^2^ Biocenter Oulu, University of Oulu, 90220 Oulu, Finland; ^3^ Center for Drug Discovery, Baylor College of Medicine, Houston, TX 77030, USA; ^4^ Department of Pathology & Immunology, Baylor College of Medicine, Houston, TX 77030, USA

**Keywords:** Implantation, Collagen, Non-linear microscopy, Second-harmonic generation, Third-harmonic generation, Smad

## Abstract

Effective interplay between the uterus and the embryo is essential for pregnancy establishment; however, convenient methods to screen embryo implantation success and maternal uterine response in experimental mouse models are currently lacking. Here, we report 3DMOUSEneST, a groundbreaking method for analyzing mouse implantation sites based on label-free higher harmonic generation microscopy, providing unprecedented insights into the embryo–uterine dynamics during early pregnancy. The 3DMOUSEneST method incorporates second-harmonic generation microscopy to image the three-dimensional structure formed by decidual fibrillar collagen, named ‘decidual nest’, and third-harmonic generation microscopy to evaluate early conceptus (defined as the embryo and extra-embryonic tissues) growth. We demonstrate that decidual nest volume is a measurable indicator of decidualization efficacy and correlates with the probability of early pregnancy progression based on a logistic regression analysis using *Smad1/5* and *Smad2/3* conditional knockout mice with known implantation defects. 3DMOUSEneST has great potential to become a principal method for studying decidual fibrillar collagen and characterizing mouse models associated with early embryonic lethality and fertility issues.

## Teaser

**Summary:** This 3D imaging method provides visual and quantitative means to assess decidual fibrillar collagen deposition, decidualization efficacy, conceptus size, and theoretical prediction of early pregnancy progression in mouse models.

## INTRODUCTION

Ongoing efforts continuously strive for a greater understanding of physiology, gene function and underlying mechanisms of human diseases. Mouse models are extensively created and used by individual research groups and national and international consortia to address these questions (Meehan et al., 2017). Accumulating data show that ∼30% of the single-gene knockout mice are embryonically or perinatally lethal (Adams et al., 2013; Dickinson et al., 2016; Muñoz-Fuentes et al., 2018) and, notably, ∼45% of the lethal phenotypes display lethality before embryonic day (E) 9.5 (Cacheiro et al., 2020; Dickinson et al., 2016). Furthermore, early embryonic lethality may occur when the ablation of maternal gene function or maternal disease conditions adversely impact reproductive organ function, implantation, decidualization or early embryo development (Chang et al., 2018; Kelleher et al., 2021; Kriseman et al., 2019; Namiki et al., 2018; Tang et al., 2022; Wang et al., 2015). Investigating early mouse embryonic stages (E4.5-E8.5) presents challenges, as both the embryo and the uterus are interdependent and therefore simultaneous studies of both are needed to holistically evaluate embryo–uterine interactions. Many studies on early implantation sites still rely on traditional, labor-intensive two-dimensional (2D) microtome sectioning of paraffin-embedded tissue yielding only a few tissue sections for future analysis – limitations that have also been noted by others (Arora et al., 2016). Accordingly, three-dimensional (3D) volumetric imaging is a compelling option for analyzing implantation sites and embryo morphology. Optical projection tomography (Dickinson et al., 2016), optical coherence tomography (Scully and Larina, 2022), micro-computed tomography (Dickinson et al., 2016; Ermakova et al., 2018; Wong et al., 2012) and light sheet microscopy (Kagami et al., 2017), although broadly used tools to analyze embryo development after E9.5, are more limited in earlier implantation site studies owing to insufficient resolution or laborious manual work to extract numerical data. It is also worth noting that many currently used 3D imaging approaches, even if applicable on embryonic stages before E9.5, are most often based on imaging isolated embryos (Dickinson et al., 2016; Ichikawa et al., 2014; Udan et al., 2014) and therefore offer no insight into the maternal response. High-frequency ultrasound imaging enables pregnancy monitoring in living animals and allows the retrieval of data about the number and volume of early implantation sites from E5.5 onwards (Peavey et al., 2017). Although this would be sufficient information for some studies, in cases when the intricate formation of the embryo–uterine interaction is in question, the resolution of this technique is still a limiting factor. In turn, 3D confocal, multiphoton and light sheet microscopy of immunostained whole implantation sites have provided an intriguing way to view the interaction between the embryo and the uterus in early pregnancy (Arora et al., 2016; Yuan et al., 2018); however, such analyses are dependent on antibody functionality and the success of time-consuming immunostaining, or on expensive genetic labeling with fluorescent markers. Thus, there is an immediate need for label-free, high-resolution, nondestructive assessment of early mouse implantation sites and the embryo–uterine relationship in early pregnancy.

A successful pregnancy in mice starts with a competent blastocyst implanting into the antimesometrial side of the uterine lumen at E4.5, which forms into an implantation chamber (Madhavan et al., 2022; Maurya et al., 2021). By implantation, the blastocyst has evolved into the embryo and extra-embryonic tissues, together called the conceptus (Hyun et al., 2020; Spencer and Bazer, 2004; Thowfeequ and Srinivas, 2022). Simultaneously, uterine endometrial fibroblast-like cells differentiate into decidual cells forming the decidua, which has crucial roles in supporting early embryo growth, such as providing a physical scaffold for the embryo before placental formation (Mori et al., 2016). Decidualization initiates extensive morphological changes around the implantation chamber, including enhanced deposition and remodeling of collagen-rich extracellular matrix (Favaro et al., 2014; Zorn et al., 1986). Collagen fibrils rapidly thicken and rearrange around the decidual cells (Alberto–Rincon et al., 1989; Carbone et al., 2006; Zorn et al., 1986). These thick fibrils are produced exclusively after implantation (Alberto–Rincon et al., 1989; Teodoro et al., 2003; Zorn et al., 1986), and have been identified as fibrillar collagen types I, III, and V (Spiess and Zorn, 2007; Spiess et al., 2007; Teodoro et al., 2003). Although the enhanced deposition of collagen upon decidualization has long been acknowledged, the biological function and the 3D organization of this collagen accumulation remain inadequately studied.

Advancements in label-free non-linear imaging technology, namely higher harmonic generation microscopy, provide great tools to study fibrillar collagen, which has a non-centrosymmetric structure and therefore intrinsically creates strong second-harmonic generation (SHG) signals (Aghigh et al., 2023). Fibrillar collagen is by far the most abundant biological structure detected with SHG imaging; only a few other structures, such as myosin and microtubules, have similar properties (Aghigh et al., 2023; Campagnola et al., 2002; Strupler et al., 2007). Complementary to SHG, third-harmonic generation (THG) mainly occurs at water–lipid and water–protein interfaces in tissues (Weigelin et al., 2016), providing a broad range of structural information. In contrast to fluorophore-based imaging, exogenous labels are not required in SHG/THG imaging, and both SHG and THG are free from photobleaching and have negligible phototoxic effects on the sample, rendering them well suitable for deep tissue imaging (Aghigh et al., 2023; Campagnola, 2011; Campagnola et al., 2002; Weigelin et al., 2016). Despite these technological developments, there are no reports, to the best of our knowledge, of using higher harmonic generation microscopy to study chemically cleared implantation sites in early mouse pregnancy.

Here, we present a pioneering method named 3D Microscopy for Optically cleared Unlabeled implantation Site Evaluation using Second and Third harmonic generation (3DMOUSEneST). 3DMOUSEneST is used to visualize and quantify the uterine decidualization reaction and conceptus growth based on label-free SHG and THG scans of intact, chemically cleared mouse uteri. We demonstrate that decidual collagen forms a 3D structure, termed the ‘decidual nest’, that offers a measurable readout of the uterine decidual response and enables assessment of decidualization efficacy and the probability of early pregnancy progression. Strikingly, the 3DMOUSEneST method enables (1) comprehensive imaging of the conceptus and the decidua, (2) quantitative evaluation of decidualization efficacy and (3) overall increased data output per specimen by providing data on both the conceptus and the uterus simultaneously and by permitting sample repurposing for other downstream applications. We envision that 3DMOUSEneST will have widespread practical applicability in characterizing the continuously increasing number of transgenic mouse models.

## RESULTS

### Decidual fibrillar collagen detected by SHG reveals a novel 3D structure named the decidual nest

**Fig. 1.**
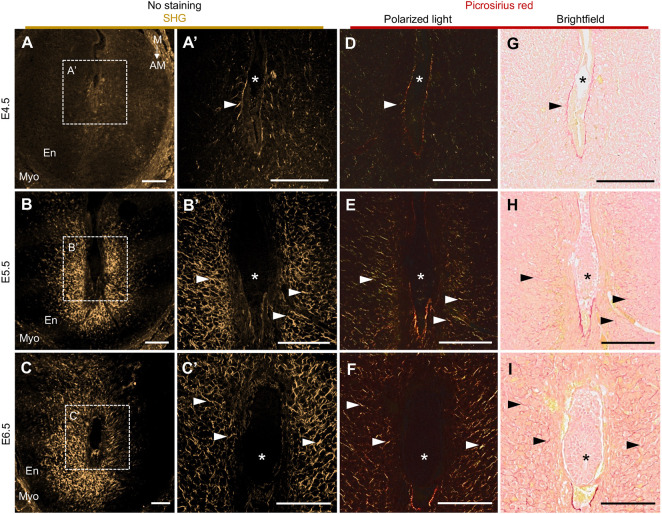
**Decidual fibrillar collagen can be detected with second-harmonic generation microscopy.** (A-C′) SHG signal (gold) of representative paraffin-embedded implantation site cross-sections at E4.5-E6.5. A′,B′,C′ show magnification of boxed areas in A,B,C, respectively. (D-I) After SHG imaging, the same implantation site cross-sections shown in A-C′ were stained with Picrosirius red and imaged using both polarized light microcopy (D-F) and standard light microscopy (G-I). Arrowheads point to examples of matching fibrillar collagen fibers detected with both Picrosirius red staining and SHG imaging. Asterisks mark the embryos. AM, antimesometrial; E, embryonic day; En, endometrium; M, mesometrial; Myo, myometrium; SHG, second-harmonic generation. E4.5 *n*=5, E5.5 *n*=5, and E6.5 *n*=8 SHG-scanned implantation sites and one subsequently Picrosirius red-stained implantation site/embryonic day. Scale bars: 200 µm.

Considering the abundance of newly deposited fibrillar collagen in decidualized uteri (Alberto-Rincon et al., 1989; Carbone et al., 2006; Favaro et al., 2014; Teodoro et al., 2003; Zorn et al., 1986) and the importance of decidualization for healthy embryo growth (Mori et al., 2016), we hypothesized that decidual fibrillar collagen could be detectable using SHG and that this could open new avenues in studying the uterine response to implantation. Initially, we used a multiphoton microscope to scan E4.5-E6.5 paraffin-embedded implantation site sections and detected a strong SHG signal in the decidual regions at E5.5 and E6.5 (Fig. 1A-C′). The SHG signal surrounded the E5.5 and E6.5 embryos in a nest-like manner, being more prominent on the antimesometrial side (Fig. 1B,C). The myometrium also showed a strong SHG signal at all studied timepoints (Fig. 1A-C). Sectioned tissues were scanned still embedded in paraffin, as paraffin did not interfere with SHG signal collection (Fig. S1). To validate that the observed SHG signal indeed corresponds with fibrillar collagen, we subsequently stained the same tissue sections with Picrosirius red (Fig. 1D-I). Picrosirius red staining is used to detect primarily fibrillar collagen when imaged with light microscopy, and it is highly specific for fibrillar collagen when imaged with polarized light (Junqueira et al., 1979; Lattouf et al., 2014). SHG signal and Picrosirius red staining have been shown to substantially colocalize in, for example, kidney and pancreatic tissues (Drifka et al., 2016; Strupler et al., 2007). Following implantation, the stromal endometrial cells gradually differentiate into decidual cells, which deposit enhanced levels of thick collagen fibrils (Favaro et al., 2014), corresponding with the progressively increasing levels of both SHG signal and Picrosirius red staining we observed around the implantation chamber from the time of implantation at E4.5 to decidualization at E5.5 and E6.5 (Fig. 1A-I). Importantly, we observed that Picrosirius red staining markedly correlated with the SHG signal (Fig. 1A′,B′,C′,D-I), verifying that SHG imaging can be used to detect decidual fibrillar collagen.

**Fig. 2.**
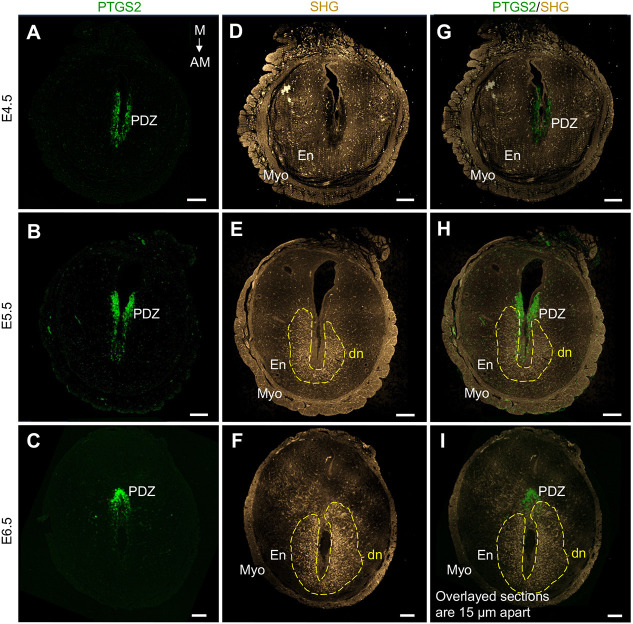
**Second-harmonic generation signal does not colocalize with the primary decidualization zone.** (A-C) Representative PTGS2 immunostaining (green) of paraffin-embedded implantation site cross-sections at E4.5 (A), E5.5 (B) and E6.5 (C), showing the primary decidualization zone (PDZ). (D-F) SHG signal (gold) from consecutive tissue sections 15 µm apart. The strongest SHG signal in the decidual area is outlined with yellow dashed lines. (G-I) Overlay of the PTGS2 staining with the SHG signal shows that the SHG signal does not significantly overlap with the PDZ. AM, antimesometrial; dn, decidual nest; E, embryonic day; En, endometrium; M, mesometrial; Myo, myometrium; PTGS2, prostaglandin-endoperoxide synthase 2; SHG, second-harmonic generation. *n*=4 implantation sites/embryonic day. Scale bars: 200 µm.

**Fig. 3.**
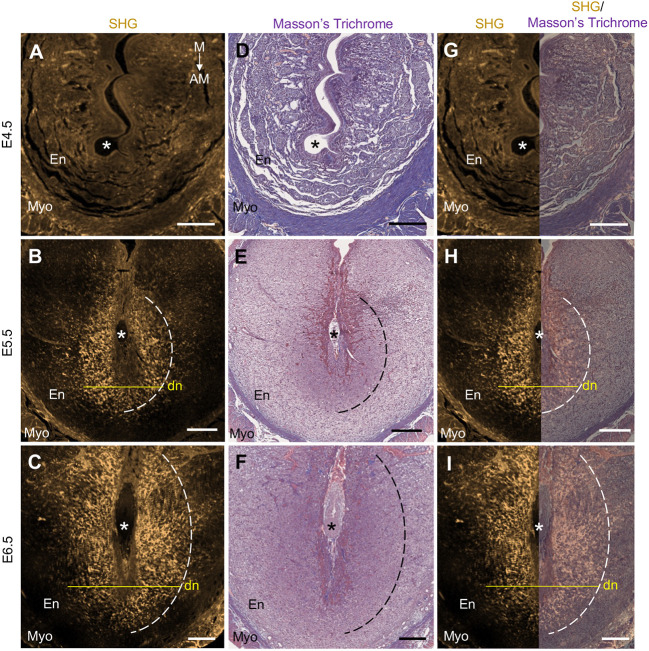
**Strong second-harmonic generation signal corresponds with the secondary decidualization zone from E5.5 onwards.** (A-C) Paraffin-embedded E4.5-E6.5 implantation site cross-sections were scanned with a multiphoton microscope showing strong SHG signal (gold; outlined with white dashed curves) in the decidual region. (D-F) The same sections were then stained with Masson's Trichrome to visualize readily differentiated decidual cells indicative of the secondary decidualization zone (SDZ), outlined with black dashed curves. The enlarged decidual cells appear as dark purple at E5.5 (E) and E6.5 (F) in comparison with the adjacent, non-decidualized endometrial stromal cells in lighter purple. Collagen stained in blue is not prominent at this magnification. (G-I) SHG signal overlayed with the Masson's Trichrome staining shows that the SHG signal conforms with the borders of the SDZ. Asterisks mark the embryos and yellow solid lines the decidual nests. AM, antimesometrial; dn, decidual nest; E, embryonic day; En, endometrium; M, mesometrial; Myo, myometrium; SHG, second-harmonic generation. *n*=3 implantation sites/embryonic day. Scale bars: 200 µm.

Decidualization proceeds in two distinct regions: the primary decidualization zone (PDZ) and the secondary decidualization zone (SDZ). The PDZ emerges immediately after implantation directly around the implantation chamber, whereas the SDZ gradually forms from the more outer proliferating decidual cells and expands from the antimesometrial to the mesometrial side (Favaro et al., 2014; Maurya et al., 2021). To further characterize the SHG signal in relation to the decidual regions, we compared the SHG signal in E4.5-E6.5 implantation sites with different decidual stainings. Immunostaining of prostaglandin-endoperoxide synthase 2 (PTGS2), a known PDZ marker (Chakraborty et al., 1996), revealed that the decidual SHG signal does not considerably colocalize with the PDZ (Fig. 2). On the contrary, based on Masson's Trichrome staining that allows morphological identification of mature, enlarged, collagen-encapsulated decidual cells from the non-differentiated endometrial cells, we could conclude that the decidual SHG signal from fibrillar collagen correlates well with the SDZ (Fig. 3).

**Fig. 4.**
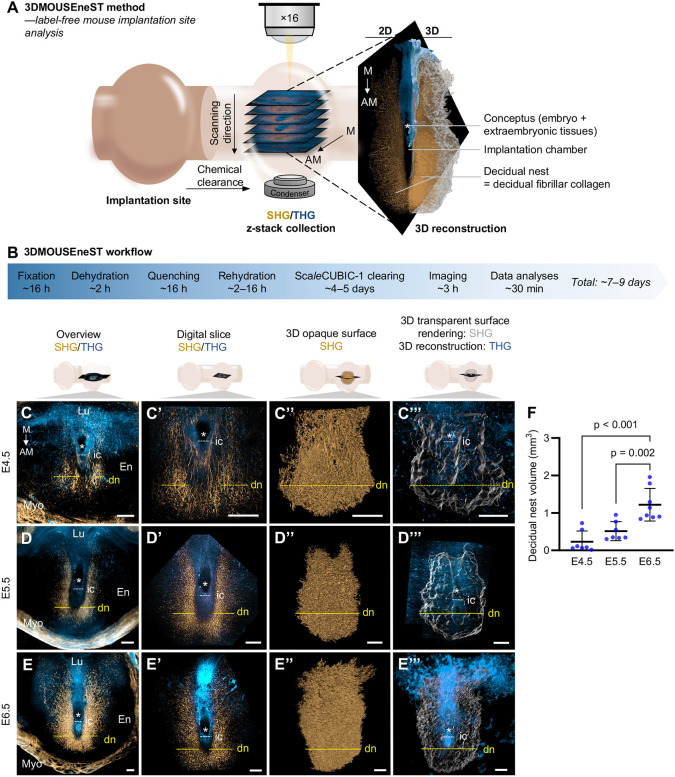
**Analysis of early mouse implantation sites in 3D with higher harmonic generation microscopy.** (A) A schematic summarizing the 3DMOUSEneST method and the structures of implantation sites detected with higher harmonic generation. Whole, chemically cleared implantation sites were scanned stepwise (*z*-stack collection) using a multiphoton microscope, detecting SHG (gold) and THG (blue) signals. 3D sample reconstruction enabled the viewing of multi-angular 2D digital tomographic cross-sections (digital slices) within the scans as well as 3D surface rendering of analyzed structures. SHG detects decidual fibrillar collagen, which was observed to form a distinct nest-like structure, coined the ‘decidual nest’ (gold; transparent surface rendering in gray), around the implantation chamber and the conceptus. The conceptus (asterisk), consisting of the embryo and the extra-embryonic tissues, is encapsulated by the THG signal (blue). (B) A workflow for the different steps of the 3DMOUSEneST method. Estimated time for each step includes both hands-on work and passive waiting gaps. Scanning and analysis times are per one sample. (C-E‴) E4.5-E6.5 implantation sites were analyzed with the 3DMOUSEneST method. Overview scanning with SHG (gold) and THG (blue) at E4.5 (C), E5.5 (D), and E6.5 (E) provided a comprehensive examination of the implantation site composition. The same E4.5-E6.5 implantation sites were then scanned through and are shown using different visualization methods: as representative digital slices (C′,D′,E′), as opaque 3D surfaces of the decidual nests (gold; C″,D″,E″), and as transparent 3D surface renderings of the decidual nests (gray) accompanied with 3D reconstructions of the THG signal (blue; C‴,D‴,E‴). See Materials and Methods for detailed information on image preparation. (F) Decidual nest volume measurements at E4.5-E6.5. AM, antimesometrial; dn, decidual nest; E, embryonic day; En, endometrium; ic, implantation chamber; Lu, uterine lumen; M, mesometrial; Myo, myometrium; SHG, second-harmonic generation; THG, third-harmonic generation. Asterisks mark the conceptuses. Yellow dashed lines indicate the developing decidual nests and yellow solid lines the established decidual nests. White dashed lines indicate the implantation chambers. Statistical analyses were performed with one-way ANOVA followed by Tukey post hoc test. The results shown are individual values with mean±s.d. E4.5 *n*=7, E5.5 *n*=7 and E6.5 *n*=8 implantation sites. Scale bars: 200 µm (C-E‴).

We then further applied SHG multiphoton microscopy to characterize whole, optically cleared E4.5-E6.5 implantation sites in 3D to assess how uniformly decidualization occurs around the embryo, establishing the SHG imaging aspect of the herein presented 3DMOUSEneST method. Graphical summaries of the complete 3DMOUSEneST workflow and timeline and the implantation site structures discussed throughout this study are presented in Fig. 4A,B. Briefly, whole implantation sites were fixed, quenched to remove autofluorescence and cleared until transparent in Sca*l*eCUBIC-1. Cleared samples were imaged with a multiphoton microscope detecting higher harmonic generation signals, having the mesometrium–antimesometrium axis parallel to the microscope stage. Multiple images were collected stepwise across the implantation sites (Movie 1), allowing 3D reconstruction, freely rotatable digital slice selection within the reconstruction, and opaque or transparent surfaces generated over the decidual SHG signal for diverse visualization purposes (Fig. 4C-E‴; Movie 2). Details of 3DMOUSEneST sample processing, imaging and image analyses are reported in the Materials and Methods section and in the Supplementary Materials and Methods as a step-by-step protocol. For orientation within the implantation sites, we first performed overview scans encompassing a wider view of the uterus, allowing for identification of collagen distribution throughout the implantation site (Fig. 4C,D,E), and then specifically focused on the decidual collagen surrounding the implantation chamber. Following sample 3D reconstruction from the stepwise-acquired SHG images, 3D surface renderings and digital slices revealed a highly distinct structure, which we termed the ‘decidual nest’, composed of decidual fibrillar collagen surrounding the implantation chamber predominantly on the antimesometrial pole (Fig. 4A,C′,C″,D′,D″,E′,E″; Movie 2). Despite seeing minimal SHG signal in E4.5 paraffin-embedded implantation site sections (Fig. 1A,A′), scanning whole, chemically cleared implantation sites in 3D proved more powerful in detecting fibrillar collagen accumulation at the same timepoint (Fig. 4C′,C″). By E5.5, decidualization had advanced remarkably, as evidenced by well-formed decidual nests (Fig. 4D′,D″), and at E6.5 they extended towards the mesometrial pole (Fig. 4E′,E″). Transparent surface rendering of decidual nests additionally allowed for visualization through the nest (Fig. 4C‴,D‴,E‴; Movie 2). To comprehend decidual nest dynamics, decidual nest volumes were calculated from the surface renderings at E4.5-E6.5. The volumes increased significantly corresponding to decidualization progression (Fig. 4F). To our knowledge, this is the first report of the 3D organization of decidual fibrillar collagen in mice.

**Fig. 5.**
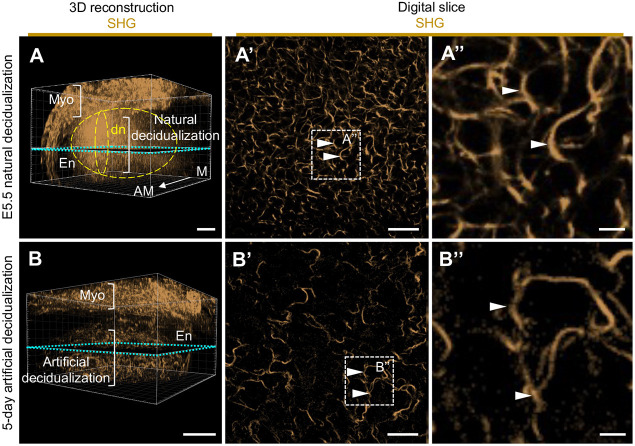
**Fibrillar collagen in natural decidualization has orderly organization whereas artificial decidualization fibrils are distributed sporadically.** (A) 3D reconstruction of the SHG signal (gold) from an E5.5 implantation site 3DMOUSEneST scan, showing natural decidualization (the decidual nest). (A′,A″) A digital slice from the decidual nest (shown in A) depicts an orderly fibrillar collagen arrangement around decidual cells (arrowheads). (B) 3D reconstruction of SHG signal (gold) from a 5-day artificially decidualized uterus. (B′,B″) A digital slice showing sporadic fibrillar collagen arrangement in artificial decidualization (arrowheads). Turquoise dashed boxes in 3D reconstructions show the plane from where the digital slices originate. White dashed boxes mark magnified digital slice areas. Yellow dashed circles indicate the decidual nest area. Brackets indicate myometrium and decidualized areas. AM, antimesometrial; dn, decidual nest; E, embryonic day; En, endometrium; M, mesometrial; Myo, myometrium; SHG, second-harmonic generation. *n*=5 natural implantation sites and *n*=4 artificially decidualized uterine horns. Scale bars: 200 µm (A,B); 50 µm (A′,B′); 10 µm (A″,B″).

To even further characterize decidual fibrillar collagen dynamics, we used SHG scanning to compare natural decidualization with artificial decidualization, which is a widely used method to study decidualization dynamics in mouse models. Naturally decidualized uteri at E5.5 displayed an orderly, woven-like collagen fibril pattern around the decidual cells (Fig. 5A-A″). In contrast, in artificially decidualized uteri, collagen fibril distribution was sporadic and did not follow a particular pattern (Fig. 5B-B″). In cases of natural decidualization, the embryo was clearly the focal point around which the collagen fibrils organized to form the supportive collagen scaffold, decidual nest (Fig. 5A-A″). Conversely, in artificial decidualization, the sporadic collagen fibrils did not form a distinguishable 3D collagen structure characteristic for natural decidualization (Fig. 5B-B″).

### THG microscopy enables characterization of the conceptus within an intact uterus

**Fig. 6.**
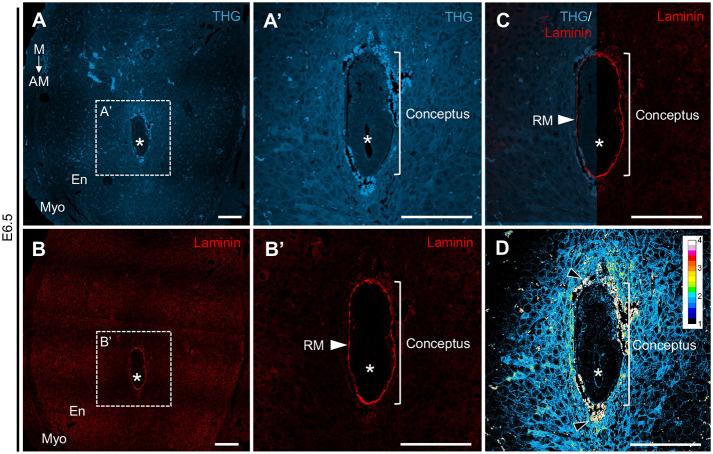
**THG imaging can be used to define the conceptus.** (A,A′) A representative E6.5 implantation site cross-section was first scanned with a multiphoton microscope using THG (blue). Note the apparent THG signal intensity differences of the surrounding uterine tissue and the conceptus. (B,B′) The same section was then immunostained with a laminin antibody (red) and imaged with a confocal microscope. Laminin is used as a marker for the conceptus-encapsulating Reichert's membrane (RM; white arrowhead). Dashed boxes in A and B indicate the magnified area shown in A′, B′, C and D. (C) Laminin immunostaining partially overlayed with the THG signal aligns with the boundary of the brighter THG signal of the surrounding tissue and the conceptus (bracket). (D) Ratio map of mean intensity fold change in relation to conceptus area intensity. The whole image was divided and normalized by the mean conceptus area intensity value. The strongest THG signal comes from the uterine tissues surrounding the conceptus, which itself has a dimmer THG signal (mean ratio of surrounding tissue to conceptus area intensity was 1.58±0.03, mean±s.d.). Red blood cells (black arrowheads) in close proximity to the conceptus were excluded from the intensity measurements. The embryo is marked with an asterisk. AM, antimesometrial; E, embryonic day; En, endometrium; M, mesometrial; Myo, myometrium; THG, third-harmonic generation. E6.5 *n*=3 implantation sites. Scale bars: 200 µm.

In addition to analyzing decidual fibrillar collagen with SHG, we explored the THG signal (Weigelin et al., 2016) to potentially provide more information on the anatomical structures of the implantation sites. THG imaging of cultured peri-implantation age mouse embryos has been described (Thayil et al., 2011), but its applicability in imaging whole implantation sites has not been reported. We hypothesized that we could use THG to image the conceptus, thus incorporating the THG imaging part into the 3DMOUSEneST method (Fig. 4A). Interestingly, we noticed a strong THG signal coinciding with the surrounding endometrial tissues, whereas the presumed conceptuses had notably weaker THG signals. As a result of avoiding overexposing the uterine tissue, the conceptuses appear as dark ‘hollows’ delineated by a bright THG signal (Fig. 4A,C,C′,C‴,D,D′,D‴,E,E′,E‴; Movie 1). To identify the boundary between the bright surrounding THG signal and the conceptus, we first scanned E6.5 implantation site sections with THG and then immunostained the same sections for laminin, an established marker for the specialized basement membrane called the Reichert's membrane encapsulating the conceptus (Bondarenko et al., 2023; Futaki et al., 2019; Matsuo et al., 2022; Ueda et al., 2020). Overlay of the laminin staining with the THG signal confirmed that a stronger THG signal of the endometrial tissue and the implantation chamber surrounds the weakly THG-emitting conceptus lined with a laminin-positive Reichert's membrane (Fig. 6A-C). To explore this in greater detail, we quantified the mean THG intensity of the conceptus and the surrounding tissue in the aforementioned E6.5 sections. Strongly THG-emitting red blood cells adjacent to the conceptus were excluded from the analyses. The surrounding tissue had a 1.58-fold stronger THG signal mean intensity compared with the conceptus area mean intensity (mean ratio 1.58±0.03, *n=*3). A representative fold-change ratio map image of the intensity changes between the conceptus and the surrounding tissue illustrates the difference in THG intensities in different locations (Fig. 6D). Taken together, THG imaging provides a convenient way to obtain structural context in the 3DMOUSEneST scans and enables identification of the conceptus based on differences in signal intensity of the different tissue compartments, making the conceptus appear as a dark ‘hollow’ within the 3D reconstructions (Fig. 4A).

**Fig. 7.**
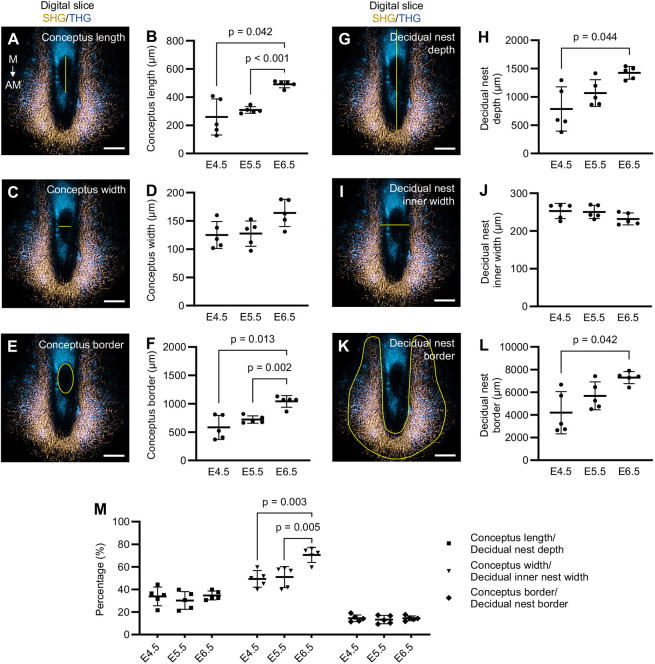
**Digital slices enable measurements of the conceptus and decidual nest growth dynamics in early pregnancy.** (A-M) Measurements derived from digital slices of the widest point of 3DMOUSEneST implantation site scans of WT mice at E4.5-E6.5. (A-F) Conceptus measurements using THG signal (blue): length (A,B), width (C,D) and border (E,F). (G-J) Decidual nest measurements (SHG, gold): depth (G,H), inner width (I,J) and border (K,L). (M) Conceptus growth in relation to decidual nest growth in early pregnancy. All measurement locations are shown from a representative E5.5 image and are indicated with yellow lines in A,C,E,G,I,K. AM, antimesometrial; E, embryonic day; M, mesometrial; SHG, second-harmonic generation; THG, third-harmonic generation. Statistical analyses were performed with one-way ANOVA followed by Tukey post hoc test in D, J and M, and with Welch's ANOVA followed by Dunnett T3 post hoc test in B, F, H and L. The results shown are individual values with mean±s.d. *n*=5 implantation sites/embryonic day. Scale bars: 200 µm.

Notably, selecting comparable digital slices from the SHG/THG 3D scans enabled us to gain greater understanding of early conceptus growth dynamics in relation to the decidual nest. Measurements showed that conceptus length and border increase significantly during early pregnancy (E4.5-E6.5), whereas the increase in conceptus width is more prolonged (Fig. 7A-F). Similarly, decidual nest depth and border increase significantly over time, but the inner width of the decidual nest displays more subtle changes, narrowing slightly at E6.5 (Fig. 7G-L). During early pregnancy, conceptus length consistently remains at ∼33% of the depth of the decidual nest, and conceptus size continually stays at ∼15% of the size of the decidual nest, whereas conceptus width significantly increases from 50% to 70% of the decidual nest inner width, suggesting that, as the conceptus expands, the decidual nest narrows around it (Fig. 7M). In comparison with traditional tissue sectioning, multi-angular digital slices from the 3DMOUSEneST scans allowed highly accurate selection of the central part of the conceptus by providing more spatial context to follow, hence enabling precise measurements. Understanding these growth dynamics allows early identification of defective implantation sites as well as recognition of whether defects are stemming from the embryonic or from the uterine component.

**Fig. 8.**
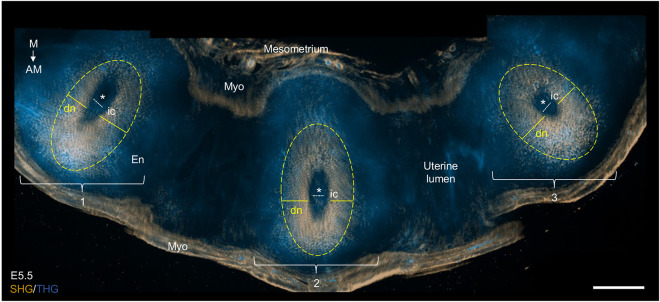
**Multiple implantation site screening with SHG/THG.** A representative wide scanning overview of three consecutive E5.5 implantation sites imaged simultaneously with SHG (gold) and THG (blue). AM, antimesometrial; dn, decidual nest; E, embryonic day; En, endometrium; ic, implantation chamber; M, mesometrial; Myo, myometrium; SHG, second-harmonic generation; THG, third-harmonic generation. Asterisks mark the conceptuses. Yellow solid lines and dashed circles indicate the decidual nests and white dashed lines indicate the implantation chambers. Numbered brackets indicate individual implantation sites. *n*=3 uterine horns. Scale bar: 500 µm.

Moreover, to demonstrate the power of 3DMOUSEneST, we report that several consecutive implantation sites in one uterine horn can be assessed simultaneously (Fig. 8). By quickly obtaining overview scans first from one plane (Fig. 8, Fig. 4C,D,E), researchers could efficiently preselect implantation sites for 3D scanning or analyses with other methods. Of note, in line with the low tissue toxicity of SHG and THG imaging (Aghigh et al., 2023; Campagnola, 2011; Campagnola et al., 2002; Weigelin et al., 2016), we confirmed that tissue samples imaged with the 3DMOUSEneST method can be used for subsequent immunostaining (Fig. S2). Accordingly, the feasibility of using tissue samples cleared with Sca*l*eCUBIC-1 for additional histological analyses has been previously demonstrated (Nojima et al., 2017; Susaki et al., 2014; Tainaka et al., 2014). The possibility for rapid preselecting of implantation sites of interest for further analysis all the more enhances the usefulness of screening embryos and decidual nests with 3DMOUSEneST.

### Decidual nest volume serves as a tool for evaluating decidualization efficacy and the likelihood of early pregnancy progression

**Fig. 9.**
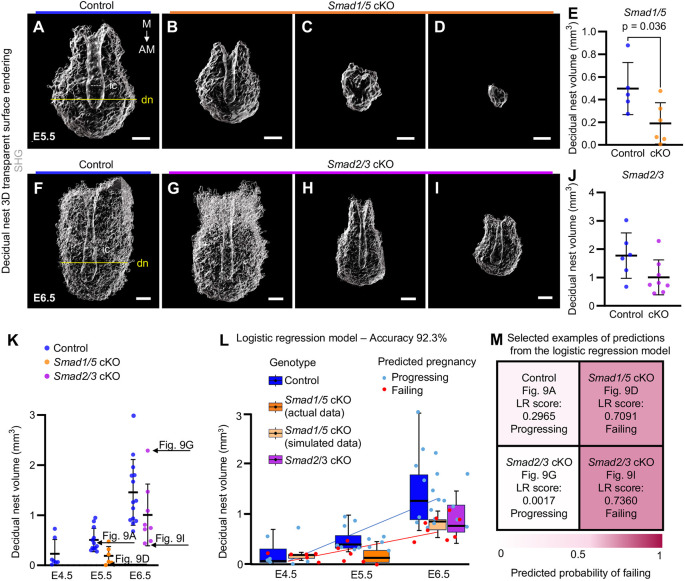
**Theoretical prediction of early pregnancy progression based on decidual nest volumes.** (A-D) Representative SHG transparent surface renderings of control and *Smad1/5* cKO decidual nests. (E) Decidual nest volumes of control (*n=*5, blue) and *Smad1/5* cKO (*n*=6, orange) mice at E5.5. (F-I) Representative SHG transparent surface renderings of control and *Smad2/3* cKO decidual nests. (J) Decidual nest volumes of control (*n*=6, blue) and *Smad2/3* cKO (*n*=8, magenta) mice at E6.5. (K) Combined decidual nest volume data from Figs 4F, 9E,J: E4.5 (*n*=7), E5.5 (*n*=12) and E6.5 (*n*=14) controls (blue), E5.5 *Smad1/5* cKO (orange), and E6.5 *Smad2/3* cKO (magenta). Arrows and figure numbers indicate samples traced through the study in Fig. 9M and Fig. S3B-K. (L) Logistic regression (LR) analysis model based on decidual nest volumes predicts implantation sites likely to progress beyond the stated embryonic day or fail, with 92.3% accuracy. Actual data for control E4.5-E6.5 (blue box), E5.5 *Smad1/5* cKO (orange box), and E6.5 *Smad2/3* cKO (magenta box) and simulated data for E4.5 and E6.5 *Smad1/5* cKO (light orange box). Blue and red dots show predictions based on the LR model for embryos likely to progress or fail, respectively. (M) Selected examples of predictions from LR model (corresponding arrows in K). AM, antimesometrial; dn, decidual nest, yellow line; E, embryonic day; ic, implantation chamber, white dashed line; M, mesometrial; SHG, second-harmonic generation. Scale bars: 200 µm (A-D); 300 µm (F-I). Statistical analyses were performed with unpaired two-tailed Student's *t*-test (E,J). The results are shown as individual values with mean±s.d. (E,J,K) or as box plots (median, horizontal line; 25%-75% interquartile range, box; 95% confidence interval, whiskers; L).

Successful implantation and decidualization mark the first major checkpoints required for a pregnancy to progress. Defects in decidualization can result in recurrent implantation failure and pregnancy loss, compromising pregnancy outcomes (Ticconi et al., 2021). To demonstrate how decidual nest volumes could be applied in determining decidualization efficacy, we present data from two mouse lines with reproductive tract-specific conditional deletions of Smad transcription factors that undergo most embryonic lethality at different times during pregnancy. Firstly, we chose *Smad1/5* conditional knockout (cKO) mice that are severely subfertile with impaired implantation and altered decidual development (Tang et al., 2022). Although implantation site count and sizes in *Smad1/5* cKO females are comparable with controls at E5.5, their numbers plummet by E6.5 (Tang et al., 2022). In our analysis of E5.5 *Smad1/5* cKO implantation sites, some decidual nests were comparable with controls in shape (Fig. 9A,B); however, most of the samples had poorly developed decidual nests and shallow implantation chambers (Fig. 9C,D; Movie 3). Overall, decidual nest volumes of *Smad1/*5 cKO mice were significantly smaller than those of controls (Fig. 9E). Secondly, we chose *Smad2/3* cKO mice that are infertile (Kriseman et al., 2019). *Smad2/3* cKO mice have a trend towards fewer and smaller implantation sites already during early pregnancy (E5.5 onwards) followed by the appearance of hemorrhagic implantation sites and substantial embryo lethality by E10.5, suggesting poor decidualization and placentation (Kriseman et al., 2019). We analyzed implantation sites from E6.5, a time when lethality starts to appear, and scanning the entire decidual nest was still technically possible. Our data showed that *Smad2/3* cKO E6.5 decidual nest volumes ranged from comparable with controls to smaller than controls (Fig. 9F-J; Movie 4), in line with reported variability in embryo lethality timepoints.

Next, to get a more comprehensive view, we plotted all analyzed decidual nest volumes of different ages and genotypes on the same graph (Fig. 9K). We observed that, when combined, all control decidual nest volumes increased over time but the mean cKO decidual nest volumes fell below those of the control, suggesting a correlation between decidual nest volume and pregnancy progression. To further test this, we fitted a logistic regression (LR) model with our own (controls E4.5-E6.5, *Smad1/5* cKO E5.5 and *Smad2/3* cKO E6.5) and computer-simulated (*Smad1/5* cKO E4.5 and E6.5) data of decidual nest volumes (Fig. 9L). According to the LR model, decidual nest volumes associate with the potential to progress to the next pregnancy day or fail with 92.3% accuracy (Fig. 9L), area under the receiver operating characteristic curve (ROC-AUC) 0.925 (Fig. S3A) and precision-recall score (PRC) 0.9199. As the LR model was trained to recognize the *Smad1/5* cKO implantation sites (imminently failing), it generated a score predicting embryonic lethality based on decidual nest volume. Hence, the lower the score, the higher the progressing probability, 0.5 being the cut-off value (Fig. 9M). We selected examples from the LR analysis representing implantation sites likely to progress further or fail (Fig. 9M) and traced them through our analyzed data to evaluate predictions (Fig. S3B-K). For example, an E5.5 control sample with LR score 0.2965 was scored as ‘progressing’ and an E5.5 *Smad1/5* cKO sample with LR score 0.7091 as ‘failing’ (Fig. 9M), in line with the expected phenotypes. Two *Smad2/3* cKO samples were assigned a LR score 0.0017, ‘progressing’ and 0.7360, ‘failing’ (Fig. 9M), respectively, again matching with the observed phenotype heterogeneity at E6.5 and allowing early detection of implantation sites that lag behind and likely would have stopped progressing completely. Importantly, our results revealed that implantation sites with high LR scores predicted as ‘failing’ have not only smaller decidual nest volume but also reduced embryo length, width and border (Fig. S3F-K), confirming a correlation between decidual nest volume and embryo development. Taken together, 3DMOUSEneST scans offer measurable parameters enabling identification of decidualization efficacy and implantation sites that were likely failing or progressing from the early days of the pregnancy.

## DISCUSSION

Proper decidualization is a hallmark of progressing pregnancy (Mori et al., 2016). So far, most studies evaluating decidualization have relied on the detection of morphologically altered decidual cells with hematoxylin and eosin staining or on *in situ* hybridization or immunostaining of sectioned tissue with known decidual markers *Bmp2*, *Wnt4*, Ki-67, PTGS2, desmin or HAND2 (Chakraborty et al., 1996; Daikoku et al., 2004; Huyen and Bany, 2011; Kelleher et al., 2018, 2021; Kim et al., 2023; Lee et al., 2007; Oliveira et al., 2000; Yuan et al., 2019). Currently published data does not show the applicability of decidual markers for 3D immunostaining of whole implantation sites and, although informative for studying the endometrial environment upon implantation, immunostaining of whole implantation sites is time-consuming and requires available biomarkers and thorough antibody penetration. Our study bridges extracellular matrix biology with reproductive biology, adding new knowledge of the 3D view of the remodeling of extracellular matrix in decidualization and applying this knowledge to establish a unique 3D analysis method, 3DMOUSEneST, to study decidual development and the potential of early pregnancy progression. We demonstrate that 3D SHG imaging and volumetric analysis of the decidual nest – the result of rapid deposition and organized arrangement of fibrillar collagen by the decidual cells around the implanted embryo – can be used to conveniently quantify decidualization without any staining or genetic labeling. We present a standard growth pattern for the decidual nest during normal early pregnancy (E4.5-E6.5). Supporting these analyses, we found that, in contrast to decidualization occurring during natural pregnancy, artificial decidualization induces only scattered uterine collagen fibril deposition, and the distinct decidual nest structure does not form at all. This illustrates the key contribution of the embryo in initiating an organized decidual collagen fiber orientation compared with artificial decidualization without an embryo. Moreover, beyond being a quantifiable marker of the decidualization reaction, decidual nest volume correlates with the theoretical probability of a specific implantation site to be able to support embryo development.

Our data also convincingly show that the conceptus can be detected with the aid of the THG signal, providing an innovative method to examine its development. Although isolated early mouse embryos have been imaged before using THG (Thayil et al., 2011), we found that when imaging intact chemically cleared implantation sites at E4.5-E6.5, the uterine tissues create such strong THG signals that the conceptus itself appears as a relatively dark and distinguishable ‘hollow’. We also noted that THG imaging of non-cleared implantation site cross-sections provides cellular-level details of the conceptus. As conceptus growth and the uterine response are very much concerted in early pregnancy to ensure successful pregnancy progression, their concurrent analysis enables gaining a broader picture of the early developmental phases. Embryo–uterine crosstalk is crucial for establishing and maintaining pregnancy, from the initial attachment of the blastocyst to the uterine luminal epithelium through the embryo-induced decidualization of the uterine stroma. Although the 3DMOUSEneST method does not explicitly visualize the luminal epithelium, it is especially well suited for analyzing the uterine response to decidualization clues along with the coinciding development of the conceptus. Noteworthily, the decidual extracellular matrix is not merely a passive structure for the decidual cells to reside in – it is dynamically involved in cellular processes mediating cell proliferation, differentiation and survival, which in turn affects how the decidual cells act towards the embryo (Favaro et al., 2014). Emphasizing the power of higher harmonic generation microscopy in early implantation site analysis, the combined data from SHG and THG imaging offers a convenient way for synergistic investigations. The ratios we calculated from the conceptus and decidual nest measurements in normal, early pregnancy demonstrate that there are quantifiable ‘norm’ ratios of the conceptus to uterine response parameters that indicate normal development. For example, the decidual nest border is consistently ∼6.5 times larger than the conceptus border throughout E4.5-E6.5. Deviation from these ‘norm’ ratios presents a useful way to identify altered pregnancy progression and to identify whether the cause is associated with the conceptus or with the maternal decidua. It is worth noting that such measurements would be hard to perform using conventional, serially sectioned implantation sites, as obtaining representative sections from all study groups is difficult to ensure. This challenge is overcome using 3DMOUSEneST, as comparable areas are selected from a digitally created plane manually oriented within the SHG/THG 3D reconstruction as desired, eliminating variation that could arise from specimen cutting angles or section selection.

Along with providing a way to easily analyze 3D decidual development and conceptus growth together based on detecting intrinsic signals of the tissue, 3DMOUSEneST offers several practical benefits. Complementing the described SHG/THG analyses, combining 3D imaging of these intrinsic properties with whole-mount immunostaining (Arora et al., 2016; Yuan et al., 2018) and/or genetic labeling (Yuan et al., 2018) could be used for even more specific implantation site analyses, providing added value and enabling an increase of the data output per specimen. Furthermore, as whole implantation sites remain intact when scanned with 3DMOUSEneST (our data) and Sca*l*eCUBIC-1 clearance is not damaging (Nojima et al., 2017; Tainaka et al., 2014), 3DMOUSEneST specimen repurposing for histological analysis methods involving sample sectioning is possible, increasing the amount of obtained data per sample while keeping the number of required laboratory animals the same. Sufficient optical clearing of tissue samples for SHG imaging can also be achieved with reagents other than Sca*l*eCUBIC-1 (LaComb et al., 2008; Ochoa et al., 2018), expanding the usability of 3DMOUSEneST in different research laboratories. Altogether, 3DMOUSEneST is advantageous in studying *ex vivo* specimens. However, owing to the need for chemical clearing of the tissue before imaging to achieve sufficient imaging quality, it cannot be applied for *in vivo* studies and therefore its usability for longitudinal studies (i.e. following a specific implantation site throughout pregnancy) is limited. Yet, there are emerging attempts to image tissues with higher harmonic generation *in vivo*, and as the development of non-linear imaging technologies further advances, the imaging depth and clarity will likely further improve (Kučikas et al., 2023). *In vivo* SHG imaging of non-cleared mouse uteri, both non-pregnant and pregnant, has been shown possible, albeit imaging was restricted to the superficial layers of uteri, which may be sufficient for uses other than decidualization reaction studies (Schmerse et al., 2014; Zenclussen et al., 2013). Despite chemical clearing, 3D imaging of more progressed implantation sites also becomes difficult by higher harmonic generation microscopy as a consequence of current technological limitations. Nevertheless, 3DMOUSEneST is particularly well suited for imaging early implantation sites as E4.5-E6.5 implantation site sizes perfectly match the feasible imaging depth.

In addition to studying normal decidual development, we also present data on the decidual nests from two mouse models with different fertility-related phenotypes: severely subfertile *Smad1/5* cKO with imminently failing implantation sites and infertile *Smad2/3* cKO with gradually failing implantation sites (Kriseman et al., 2019; Tang et al., 2022). Our results reflect the previously published biology of these mouse models, validating the presented method and affirming its power in early implantation site studies as our SHG-derived data (decreased decidual nest volume) and THG-derived data (smaller conceptuses) of *Smad* cKO implantation sites suggest aberrant embryo development consistent with the reported pregnancy failures. By applying an LR analysis to decidual nest volumes of both control and *Smad* cKO models, we discovered that higher LR scores correlated with smaller decidual nest volumes and smaller conceptus measurements. Based on this, we suggest that decidual nest volume can serve as a reliable prognostic measurement in defining decidualization efficacy and the likelihood of early pregnancy progression to the next developmental stage. It is worth noting that the LR prediction has its limitations as it will provide only a theoretical pregnancy progression likelihood, for which *in vivo* validation is not possible as 3DMOUSEneST analyses are conducted *ex vivo* using already fixed specimens. Meanwhile, as an advantageous tool, we used computer simulation to generate E4.5 and E6.5 *Smad1/5* cKO data, a strategy useful in overcoming the challenges of obtaining samples from embryonically lethal mouse lines. All in all, our data illustrate that decidual nest volume analysis has the potential to become a valuable tool in assessing not only decidual development but also identifying *ex vivo* such implantation sites that would have most likely failed and those that would most likely have developed further in early pregnancy.

Herein, we have described the initial methodological establishment and proof-of-concept applications of 3DMOUSEneST, and we foresee widespread future applicability for the method in characterizing the roles of either maternal or embryonic genetic modifications or environmental factors for fertility. To show that the decidual nests are compromised in implantation sites of mouse models with decidual and fertility defects known to be of maternal origin we used *Smad* transgenic mice, but the method could also be applied to examine early embryo development when the maternal side is normal but the embryos are adversely affected, for example, by a genetic alteration. In these cases, imaging intact uterine horns, i.e. multiple implantation sites at once, could be an especially advantageous tool in phenotypic screening of embryonic lethality. It could enable both individual scientists and mouse phenotyping consortiums to perform streamlined early embryo phenotyping and obtain more data from the samples used. Of note, studies of genetically manipulated mice have discerned that impaired decidualization is frequently associated with placental failure, as a continuous dialogue between the embryo-derived trophoblast cells and the maternal decidua during the first days after implantation is crucial for following placentation (Hemberger et al., 2020). 3DMOUSEneST could help to identify those early decidual alterations preceding placental development. Additionally, this technique has the potential to expand our understanding of how chronic diseases affect decidualization and pregnancy outcomes, facilitating mouse model developments with corresponding disease phenotypes and evaluation of therapeutic discoveries. We also anticipate that the discovery of the decidual nest as a result of 3D reconstruction of uterine SHG signal has the potential to inspire future identification of 3D fibrillar collagen assemblies akin to decidual nests in other organs, with potential diagnostic value.

To conclude, here we present a cutting-edge technique to image whole, chemically cleared early mouse implantation sites in a 3D and label-free manner. 3DMOUSEneST provides sufficient resolution for simultaneous, convenient visualization of both the conceptus and the corresponding uterine decidualization, filling in the hitherto missing gaps in 3D imaging of the immediate uterine response to the implanted embryo and in decidualization efficacy evaluation. Given the streamlined workflow and increased data output that 3DMOUSEneST offers, we envision it to become a leading method for investigating mouse models associated with decidual defects, early embryo lethality and fertility issues. Furthermore, along with the prospective broad use of the herein described method in characterizing genetically modified and experimental mouse models, all advancements in non-linear imaging technologies are valuable in contributing to the ongoing development of novel human diagnostic tools.

## MATERIALS AND METHODS

### Mouse models and tissue collection

#### Ethics and animal care

Animal handling was conducted in accordance with Oulu Laboratory Animal Center (OULAC; University of Oulu, Oulu, Finland) and Finnish institutional animal care policies as well as the guidelines of the Institutional Animal Care and Use Committees of Baylor College of Medicine (Houston, TX, USA), which fully meet the requirements of the European Union Directive 2010/63/EU and European Convention for the protection of vertebrate animals used for experimental and other scientific purposes (ETS No. 123, appendix A) and the NIH Guide for the Care and Use of Laboratory Animals. All mice in OULAC and in Baylor College of Medicine were group-housed in specific-pathogen free conditions in individually ventilated cages under controlled environmental conditions (21°C, 50% humidity, 12 h light/12 h dark) and had access to standard pellet food and water *ad libitum*. In OULAC, the breeding experiments were conducted in conventional unit facilities in open cages.

#### Breeding and tissue collection

For wild-type (WT) breeding, mature C57BL/6NCrl or hybrid C57BL/6J/129S5/SvEvBrd mice were housed together (one male and two females per cage), and breeding was confirmed by checking for post-coital plugs the following morning, which for all gestational time points was considered as E0.5. Artificial decidualization using C57BL/6J/129S5/SvEvBrd mice was performed as previously reported (Tang et al., 2022). *Smad1/5* cKO female mice (Tang et al., 2022) were generated as reported before by crossing floxed *Smad1/5* mice with lactoferrin-*Cre* mice, and *Smad2/3* cKO female mice (Kriseman et al., 2019) by crossing floxed *Smad2/3* mice with progesterone receptor–*Cre* mice, and implantation sites were obtained by breeding with WT males as described previously (Kriseman et al., 2019; Tang et al., 2022). The female mice used in all breedings were 8-21 weeks of age. For tissue collection, mice were euthanized by carbon dioxide overdose and cervical dislocation. Pregnant and artificially decidualized uteri were dissected and fixed in 4% paraformaldehyde overnight at +4°C or in 10% formalin overnight at room temperature. Samples were washed in 1× phosphate buffered saline (PBS) and dehydrated stepwise to 70% ethanol. E4.5 and some of the E5.5 WT implantation sites were identified by injection of 1% Chicago Sky Blue dye before euthanasia (Tang et al., 2022) (the dye was washed away before scanning and did not affect the imaging).

### 2D implantation site multiphoton scanning and supporting histologic analyses

#### Sectioned tissue preparation

Briefly, fixed implantation sites were dehydrated in increasing ethanol concentrations, washed in xylene, embedded in paraffin and sectioned (5 µm) on objective slides (SuperFrost™, Plus Adhesion Microscope Slides, Epredia). For demonstrating sample repurposing after 3DMOUSEneST imaging (Fig. S2), Sca*l*eCUBIC-1 reagent was removed from a cleared implantation site by washing in 1×PBS supplemented with 1% Triton X-100 for 5 days, changing the washing solution daily, before processing for paraffin embedding.

#### Multiphoton microscopy of sectioned tissue

For imaging the SHG signal in tissue cross-sections, they were first scanned with SHG before performing any staining, as we noticed that histologic stainings interfere with SHG signal detection. Of note, we confirmed that paraffin did not markedly affect the SHG signal (Fig. S1), and therefore tissues remained embedded in paraffin during sectioned tissue SHG scans. However, paraffin emits a strong THG signal (Fig. S1), and consequently, paraffin sections were deparaffinized with xylene, rehydrated in decreasing ethanol concentrations, and mounted with Immu-Mount (Thermo Fisher Scientific) before THG signal scanning. Accordingly, when both SHG and THG signals were imaged, tissue sections were deparaffinized before imaging. In detail, all implantation site sections were scanned using an upright Nikon A1R MP+ multiphoton microscope with a tunable femtosecond laser (Coherent Chameleon Discovery) and NIS-Elements C-ER acquisition software. A CFI75 LWD 16×/0.8W DIC objective was used for all. SHG and THG signals were recorded using both episcopic (epi) and transmitted (trans) direction non-descanned detectors. Transmitted signals were collected with a Nikon A1-NDN NA 1.2W condenser. SHG and THG excitation wavelengths were both set to either 1100 or 1180 nm. In the epi direction, SHG emission was collected at 511-593 nm and in the trans direction at 495-560 nm (1100 nm) and above 560 nm long pass (1180 nm). THG emission was collected under 458 nm short pass in both the epi and trans directions. Because the mouse uterus cross-sections were too large to view in a single 16× field of view, tile scanning was carried out to encompass the entire section. The number of tiles was determined by the sample size.

#### Picrosirius red staining

Paraffin-embedded tissue sections were incubated for 1 h at 55°C, deparaffinized in xylene and rehydrated with decreasing ethanol concentrations. Sections were then stained for 1 h in Picrosirius red stain (0.1% Direct red 80, Sigma Aldrich, in saturated picric acid) and washed for 2 min in acidified water. Finally, sections were quickly dehydrated in increasing ethanol concentrations and xylene and mounted with Pertex. Stained sections were imaged with an Olympus VS200 slide scanner using brightfield and polarized light channels and UPLXAPO 40×/0.95 objective and processed using OlyVIA V4.1.1 software.

#### Masson's Trichrome staining

Paraffin-embedded tissue sections were stained using a Masson's Trichrome staining kit (Sigma Aldrich, HT15-1KT). Sections were incubated for 1 h at 55°C, deparaffinized in xylene, and rehydrated with decreasing ethanol concentrations. Sections were then stained for 10 min in Weigert's iron hematoxylin, rinsed for 10 min in running warm tap water, stained in Biebrich scarlet–acid fuchsin solution for 15 min, washed in distilled water 3 min, differentiated in phosphomolybdic acid/phosphotungstic acid solution for 20 min, stained in aniline blue solution for 7 min, and then moved to freshly prepared 1% acetic acid solution. Sections were dehydrated in increasing ethanol concentrations and xylene and mounted with Pertex. Stained sections were imaged with Hamamatsu NanoZoomer S60 digital slide scanner using 40× mode and viewed using the NDP.view2 software.

#### PTGS2 immunohistochemistry

After deparaffinization and rehydration, tissue sections were boiled in 0.01 M sodium citrate (pH 6.0) for 20 min. Following antigen retrieval, sections were blocked in 5% fetal bovine serum (FBS) and 5% goat serum for 1 h at room temperature. Primary antibody incubation using anti-PTGS2 antibody (also known as COX-2; 1:100, Cayman Chemicals, 160106) was performed overnight at 4°C and the secondary antibody incubation using Dako anti-rabbit Immunoglobulin/HRP (1:200, Dako, PO448) for 1 h at room temperature in 0.5% FBS and 0.5% goat serum. Staining was visualized using a DAB (3,3-diaminobenzidine) substrate kit (Vector Labs, SK-4100) following the manufacturer's instructions. Sections were mounted with Immu-Mount (Thermo Fisher Scientific). Stained sections were imaged with a Zeiss Axio Imager.M2 m motorized light microscope using an EC Plan-Neofluar 5×/0.16 M27 objective, Zeiss Axiocam 506 color camera, and Zen 2.3 Pro software. To merge the PTGS2 staining with a SHG image from consecutive sections of the same implantation site (15 µm apart), the DAB signal was assigned a green pseudo-color in Image J (Fiji) (Schindelin et al., 2012) and the images were overlayed in PowerPoint (Microsoft).

#### Laminin immunohistochemistry

Tissue sections deparaffinized and mounted for THG imaging as described above were incubated in 1×PBS supplemented with 0.1% Triton X-100 overnight to detach coverslips. Slides were then boiled in 0.01 M sodium citrate (pH 6.0) for 20 min. Following antigen retrieval, sections were blocked in 5% FBS and 5% goat serum for 1 h at room temperature. Primary antibody incubation with a pan-laminin antibody (1:100, Abcam, ab11575) was performed overnight at 4°C and the secondary antibody incubation using goat anti-rabbit antibody conjugated with Alexa Fluor 546 (1:1000, Invitrogen, A-11010) was carried out for 1 h at room temperature in 0.5% FBS and 0.5% goat serum. Sections were mounted with Immu-Mount (Thermo Fisher Scientific). Stained sections were imaged with a Zeiss LSM780 confocal microscope with a Plan Apochromat 20×/0.8 air objective and processed in Zen 3.5 Blue edition software. Overlay of laminin staining with THG images was carried out in PowerPoint (Microsoft).

#### THG intensity measurements from tissue sections

To measure THG intensity in implantation site sections, a region of interest (ROI) was drawn around the conceptus using ‘Freehand’ mode in Image J (Fiji) and mean gray value was measured. An ‘Oval selection’ was used to draw an ROI encompassing the surrounding endometrial tissue. The same oval ROI was used for all samples for consistency. The conceptus and adjacent maternal blood cells were excluded from the surrounding tissue mean gray value measurements. Three tissue sections/implantation site were analyzed, and average mean gray values were collected from each. Fold change was calculated by dividing the mean gray value of the surrounding tissue by the mean gray value of the conceptus. To generate the fold change ratio map image (Fig. 6D), the mean gray value of the conceptus area was measured and the whole image was divided by that value. A 16 colors look-up table was applied to the resulting fold change image, and it was scaled to show the fold change values between 1 and 4.

### 3D implantation site multiphoton scanning: the 3DMOUSEneST method

3DMOUSEneST sample processing and scanning are summarized below. A detailed step-by-step protocol for the whole 3DMOUSEneST method from sample collection to analyses can be found in Supplementary Materials and Methods.

#### 3D sample quenching and clearing

Fixed and dehydrated whole implantation sites were quenched in freshly prepared 2:1:3 ethanol:DMSO:H_2_O_2_ (30% stock solution) overnight at room temperature while rotating. After quenching autofluorescence and removal of tissue coloration, samples were washed twice for 30 min in 70% ethanol, rehydrated stepwise to 1×PBS and washed overnight at room temperature. Artificial decidualization samples were not quenched but otherwise treated the same as implantation sites. Samples were cleared using Sca*l*eCUBIC-1 reagent (Susaki et al., 2014): 25wt% urea, 25wt% Quadrol [N,N,N′N′-tetrakis(2-hydroxypropyl)ethylenediamine; Sigma-Aldrich] and 15wt% Triton X-100. Clearance was carried out with samples rotating at room temperature for at least 4 days, changing Sca*l*eCUBIC-1 daily, until samples were transparent.

#### 3D sample preparation for imaging

Cleared, whole implantation sites and artificial decidualization samples were imaged in a custom-made sample chamber. The chamber base consisted of a 24×32 mm #1 coverslip (Menzel-Gläser or Marienfeld) and a ring of Blu-Tack (Bostik) to seal the sample suspended in Sca*l*eCUBIC-1, and the assembly was covered with a 22×22 mm #1 coverslip (Menzel-Gläser). Implantation sites were imaged either in an entire horn or individually. For scanning, samples were oriented so that the mesometrium–antimesometrium axis was parallel to the microscope stage (Fig. 4A).

#### 3D multiphoton microscopy

Whole implantation site scans were acquired using a Nikon A1R MP+ multiphoton microscope using a similar microscope setup as described above in ‘Multiphoton microscopy of sectioned tissue’. SHG and THG excitation wavelength was set to 1100 nm. In the epi direction, SHG emission was collected between 511 and 593 nm, while trans SHG emission was collected between 495 and 560 nm. THG emission was collected under 458 nm short pass in both epi and trans directions. Emissions were collected on both epi and trans directions, but trans SHG and epi THG were found to give the best contrast and were used in images and analyses reported. To acquire entire decidual nest scans, tile scanning was used throughout the image stacks, with the number of tiles needed dependent on the sample and decidual nest size. A gradual laser intensity compensation was used to maintain even brightness as the *z*-direction depth increased. For WT implantation site scans, E4.5 and E5.5 images were acquired at 4 µm and E6.5 images with 5 µm steps. *Smad1*/5 and *Smad2*/*3* cKO implantation site scans were acquired using 4 µm and artificial decidualization scans using 2 µm steps. *x*×*y* pixel sizes were 0.79 µm or 1.59 µm.

### 3DMOUSEneST image processing and analyses

#### Image analysis software

All 3DMOUSEneST image processing and analyses were performed using Imaris software (Bitplane; version 9.8.0). Individual movies produced in Imaris (i.e. representative *z*-stacks and decidual nests) were combined into one view Movie files in ImageJ (Fiji). Movie annotations were added using Corel VideoStudio (2022).

#### Image processing

Multiphoton scan images (*z*-stack collection) were reconstructed into 3D automatically using the ‘Volume’ mode. SHG and THG signal is shown using the ‘MIP’ mode in Fig. 4A,C,C′,D,D′,E,E′, and Movies 1-4. In Fig. 4C″,D″,E″ ‘Normal shading’ mode was used to highlight the surface of the decidual nest, referred to as ‘opaque surface’ in Fig. 4. Thresholds for trans SHG and epi THG were set manually and optimized for each sample to view the decidual nest and outline the conceptus border and to compensate for varying imaging depths and tissue clearing. To exclude the myometrium in the representative decidual nest images and movies, the ‘Mask’ feature (within ‘Surfaces’) was used to duplicate the SHG signal that was identified in the decidual nest surface, so only the decidual nest SHG signal is displayed (Fig. 4A,C″,C‴,D″,D‴,E″,E‴, Fig. 9A-D,F-I; Fig. S2B; Movies 2-4). The transparent surface rendering option (in gray in Fig. 4A,C‴,D‴,E‴, Fig. 9A-D,F-I; Movies 2-4) was used to additionally visualize the decidual nest interior. Also, in Fig. 4A and Movie 2, scene 6, the THG signal was cropped around the implantation chamber for clearer visual representation, and in Fig. 4A, the 3D THG signal is presented with surface detail smoothing set to 15 µm and ‘Absolute Intensity’ thresholding selected.

#### Decidual nest volume measurements from 3D scans

Decidual nest surface rendering and volume measurements were done using the ‘Surfaces’ feature. Briefly, the decidual nest surface was generated using ‘Create algorithm’ for the trans direction SHG channel, with surface detail smoothing set at 15 µm and ‘Absolute Intensity’ thresholding selected. Thresholds for surface identification were set manually using the slicer view and optimized for each sample to best cover the decidual nest border. In cases where the decidual nest surface rendering touched the myometrium, the editing tool ‘Cut surface’ was used to separate the decidual nest from the myometrium. All myometrium surfaces were excluded from volume measurements. The volume of the decidual nests was measured with the ‘Statistics’ feature by selecting the decidual nest surface and choosing ‘Volume’.

#### Conceptus and decidual nest growth measurements from digital slices

To measure conceptus growth in relation to the decidual nest, trans SHG and epi THG channels were used, viewing the decidual nest and conceptus in 3D ‘Volume’ mode. First, the decidual nest was oriented so that its top (mesometrial side) was towards the top of the screen and the bottom (antimesometrial side) was towards the bottom of the screen. To measure decidual nest and conceptus dimensions, we consistently chose the digital slice from the widest, central part of the conceptus within the 3D reconstruction by using the ‘Oblique’ slicer tool in Imaris (Fig. 7). The THG signal coming from the conceptus was weaker when compared with the intense signal generated by surrounding tissues, and thus the conceptus appeared as a dark ‘hollow’ for measurements. Measurements were made using ‘Measurement points’ with only the ‘Oblique’ plane activated. Of note, the digital slice selected using the ‘Oblique’ slicer feature is not synonymous with a single image in the *z*-stack. The oblique slice is digitally created and allows for multi-angular rotation and viewing inside the 3D reconstruction.

### Statistical analyses

Normal distribution of the data was confirmed with the Shapiro–Wilk test of normality, and the homogeneity of variances was analyzed with Levene's test. Comparisons of two groups were performed using a two-tailed unpaired Student's *t*-test (equal variances) or Welch's *t*-test (unequal variances), whereas comparisons of more than two groups were performed using one-way ANOVA followed by Tukey post hoc test (equal variances) or Welch's ANOVA followed by Dunnett's T3 multiple comparisons test (unequal variances). Levene's test was performed using Origin Pro software (OriginLab), and the other statistical tests were performed using Prism 9.5.0 (GraphPad).

To build the LR model, the actual data of decidual nest volumes of control E4.5-E6.5 and *Smad1/5* cKO E5.5 implantation sites were used, whereas the corresponding E4.5 and E6.5 *Smad1/5* cKO decidual nest volume data were modeled using a truncated normally distributed model based on the decidual nest volume ratio of control (E4.5-E6.5) and *Smad1/5* cKO at E5.5. Actual and simulated data were used to train an LR model in R, with a hold-out training/testing split set at 0.75, using a generalized linear model framework with the sigmoid response. Accuracy, balanced accuracy, AUC-ROC and PRC were used as indicators of model fitting. Both class predictions and probabilities were retrieved from the trained model and confronted against ground truth. *Smad2/3* cKO data were not used for model construction.

In all graphs, data are presented as individual values with mean±s.d., except for LR data in Fig. 9L, where the box plot shows median (horizontal line), 25%-75% interquartile range (box) and 95% confidence interval (whiskers). The exact *P*-values when *P*≤0.05 are indicated in the graphs and the statistical tests used in each graph are indicated in the corresponding figure legends. At least three implantation sites from at least two pregnant female mice were used for each gestational time point in each experiment, except in Fig. 1 for the Picrosirius staining and Fig. S2 for the laminin immunostaining repurposing, in which one sample/embryonic day was stained. The exact implantation site number used in each experiment is indicated in the figure legends.

## Supplementary Material



10.1242/develop.202938_sup1 Supplementary information
