# Supplementary material for: 3DMOUSEneST: a volumetric label-free imaging method evaluating embryo–uterine interaction and decidualization efficacy
Source: Development. 2024 Aug 29;151(16):dev202938. doi: 10.1242/dev.202938 (PMC11385321; doi:10.1242/dev.202938)
Supplement: Supplementary information [file develop-151-202938-s1.pdf]

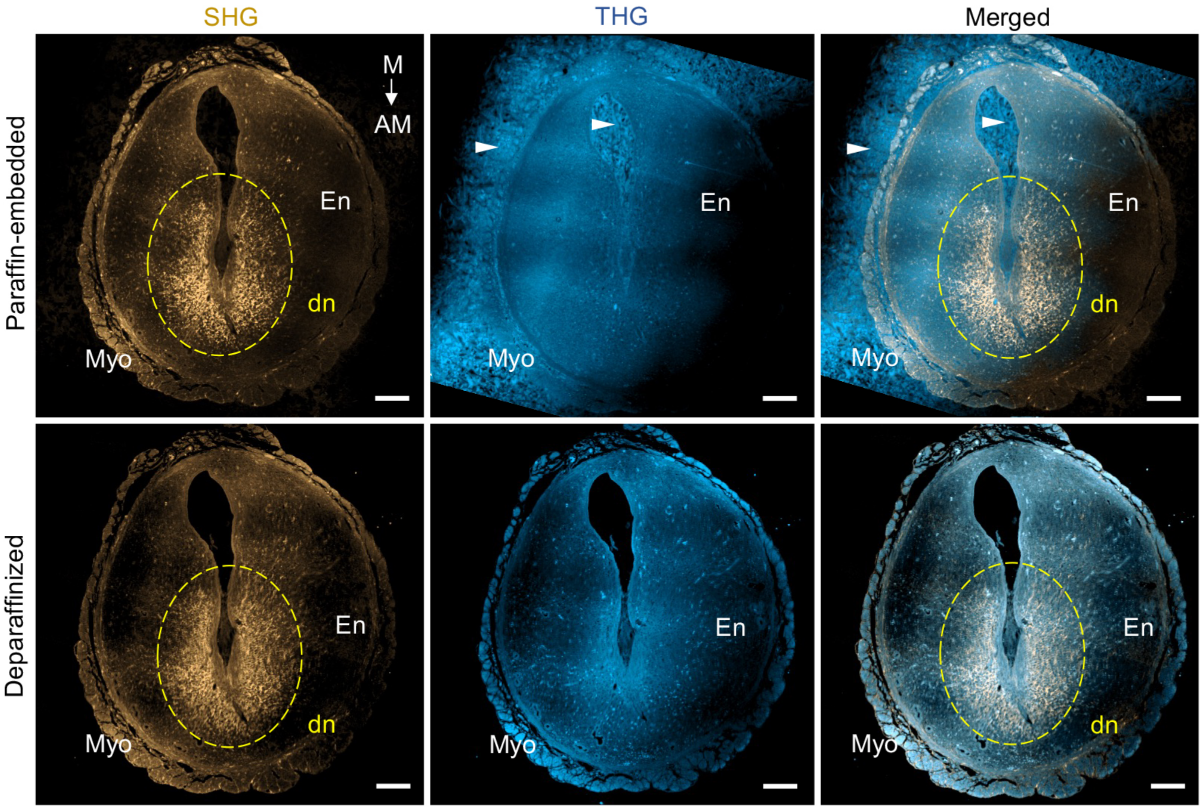

Fig. S1. Paraffin does not affect the SHG signal but interferes with the THG signal in tissue sections. Upper row: A representative E5.5 paraffin-embedded implantation site section scanned with a multiphoton microscope using higher harmonic generation, showing SHG (gold), THG (blue), and a merged image. Lower row: The same E5.5 implantation site section scanned after deparaffinization, showing SHG (gold), THG (blue), and a merged image. Arrowheads indicate paraffin-derived THG signal. AM, antimesometrial; dn, decidual nest, yellow dashed circle; E, embryonic day; En, endometrium; M, mesometrial; Myo, myometrium; SHG, second-harmonic generation; THG, third-harmonic generation. *n*=3 implantation sites. Scale bars: 200 µm.

Development • Supplementary information

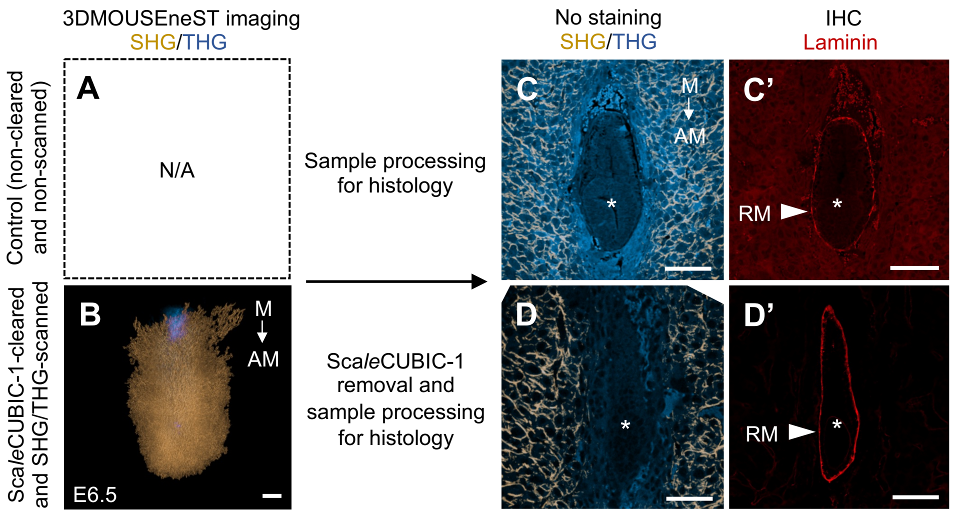

Fig. S2. Sample repurposing for immunostaining is possible after conducting the 3DMOUSEneST protocol. (A) A fixed control E6.5 implantation site sample was directly processed for paraffin embedding and sectioning without tissue clearing or SHG/THG imaging. (B) An E6.5 implantation site was first optically cleared in Sca*le*CUBIC-1 and imaged collecting SHG (gold) and THG (blue) signals (same sample as in Fig. 4E–E'''). Thereafter, Sca*le*CUBIC-1 was removed by extensive washing in 1×PBST (1% Triton X-100) and the sample was processed for paraffin embedding and sectioning. (C,D) Deparaffinized tissue cross-sections were imaged with SHG/THG microscopy to demonstrate that the intrinsic SHG and THG properties persist also after intense tissue processing. Coverslips were thereafter gently detached by overnight incubation in 1×PBST (0.1% Triton X-100). (C',D') The same tissue sections were then immunostained with laminin antibody (red) detecting the Reichert's membrane (RM; arrowheads) around the conceptus, showing that sample repurposing for immunostaining after performing 3DMOUSEneST analyses is feasible. Asterisks mark the embryos. AM, antimesometrial; E, embryonic day; IHC, immunohistochemistry; M, mesometrial; SHG, second-harmonic generation; THG, third-harmonic generation. Scale bars: 200 µm in B and 100 µm in C–D'.

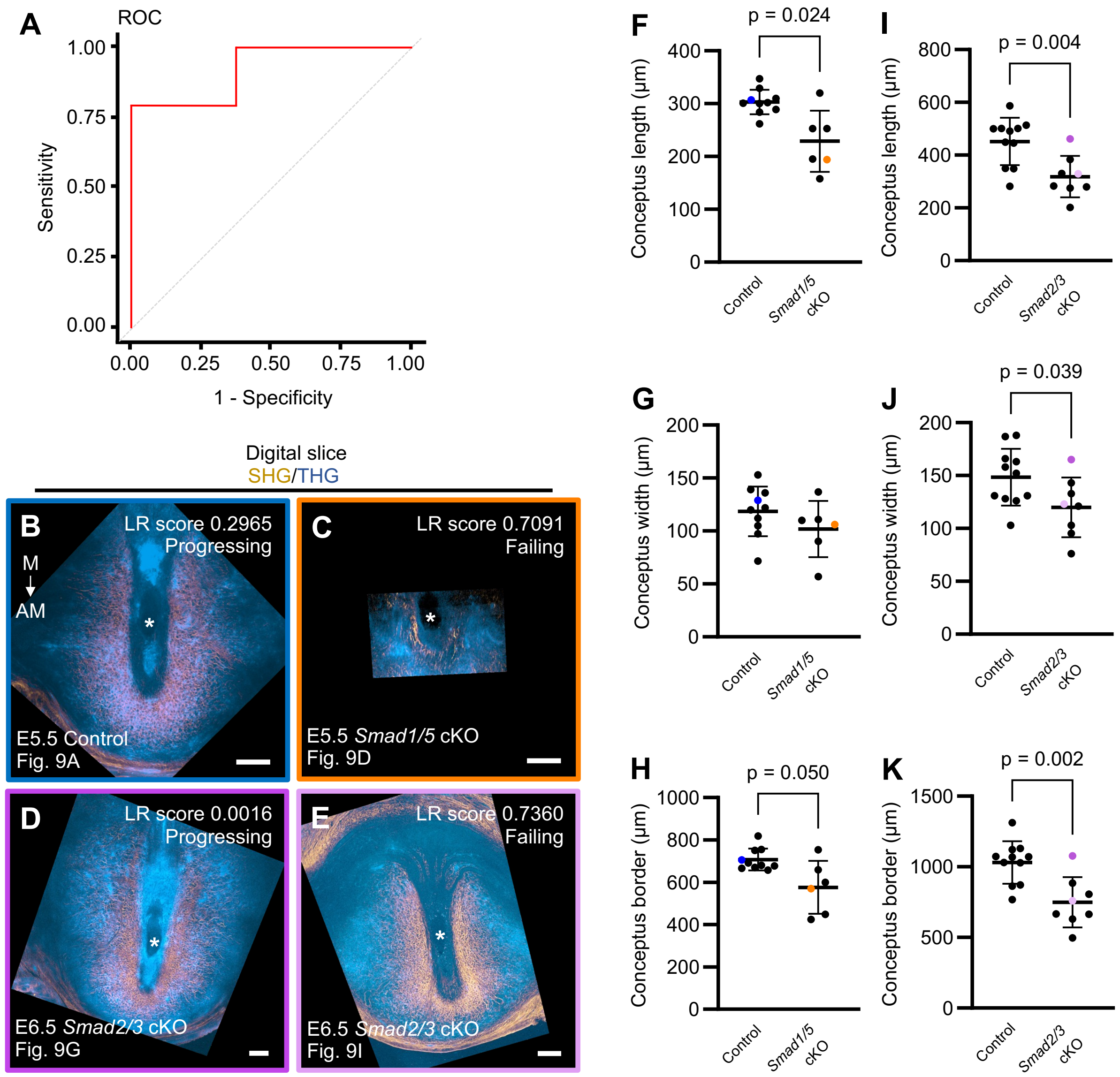

Fig. S3. ROC curve and representative implantation site images and embryo measurements that support logistic regression (LR) model accuracy. (A) ROC curve for the LR model presented in Fig. 9L. (B–E) SHG (gold) and THG (blue) digital slices of the decidual nest and conceptus with corresponding LR score, panel number for decidual nest image in Fig. 9, and prediction for pregnancy progression (progressing or failing) from selected examples of a progressing E5.5 control (B), failing E5.5 *Smad1/5* cKO (C), progressing E6.5 *Smad2/3* cKO (D), and failing E6.5 *Smad2/3* cKO (E) implantation site. (F–H) Conceptus measurements from E5.5 control and E5.5 *Smad1/5* cKO: conceptus length (F), conceptus width (G), and conceptus border (H), with selected data points corresponding to B (blue dot) and C (orange dot). (I–K) Conceptus measurements from E6.5 control and E6.5 *Smad2/3* cKO: conceptus length (I), conceptus width (J), and conceptus border (K), with selected data points corresponding to D (magenta dot) and E (light magenta dot). Asterisks mark the conceptuses. The image border color of B–E matches the dot color in graphs F–K. AM, antimesometrial; M, mesometrial. Statistical analyses were performed with unpaired Student's t-test in G and I–K and with Welch's t-test in F–H. The results are shown as individual values with mean ± sd. *n*=10 *Smad1/5* control, *n*=6 *Smad1/5* cKO, *n*=11 *Smad2/3* control, and *n*=8 *Smad2/3* cKO implantation sites. Scale bars: 200 µm (B–E).

Development • Supplementary information

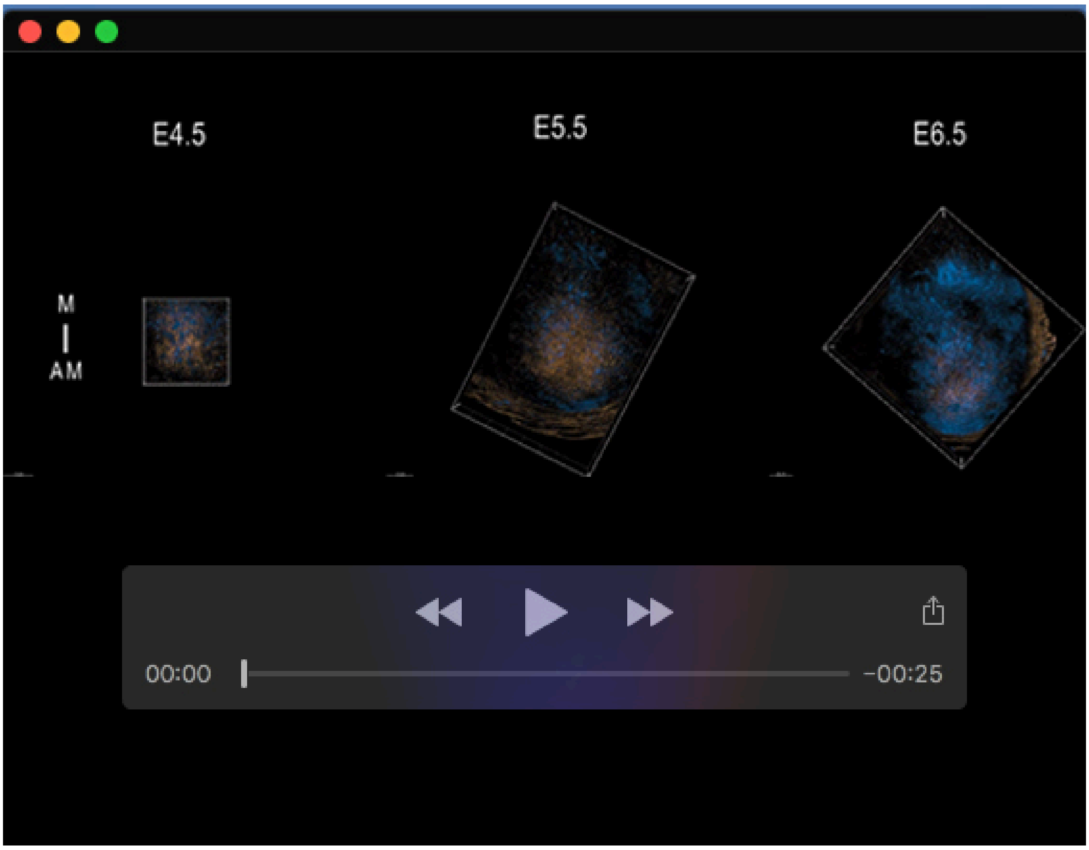

Movie 1. Representative z-stack views through WT 3DMOUSEneST implantation site scans at E4.5, E5.5, and E6.5. From left to right: z-stack images taken across WT E4.5, E5.5, and E6.5 implantation sites. SHG (gold) is visualizing fibrillar collagen of both the decidual nest and the myometrium. THG (blue) provides context to outline the conceptus (darker 'hollow' in the middle of the scans). An asterisk indicates the conceptus position, shown in the top-to-bottom z-stack series, but is excluded from the subsequent bottom-to-top z-stack series for unobstructed conceptus viewing. Scale bars for all are 300 µm. AM, antimesometrial; E, embryonic day; M, mesometrial.

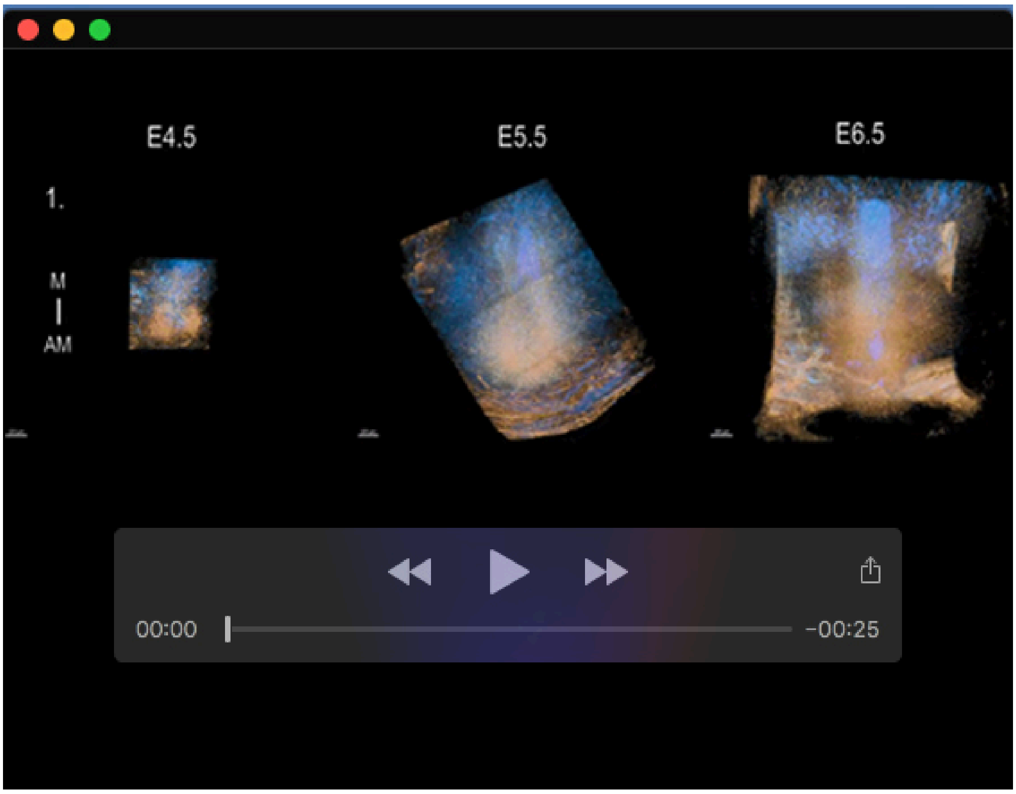

Movie 2. Multimodal views of representative WT E4.5, E5.5, and E6.5 implantation site 3DMOUSEneST scans. From left to right: E4.5, E5.5, and E6.5 WT decidual nests (SHG is shown in gold and its transparent surface rendering in grey). THG (blue) provides context to outline the conceptus (darker 'hollow'). Scale bars for all are 200 µm. The video shows the following progression:

1. Unedited scan: SHG (gold) includes part of the myometrium and the decidual nest, and THG (blue) surrounds the conceptus and shows uterine tissue context.
2. Decidual nest (SHG in gold) + tissue context (THG in blue).
3. Decidual nest (SHG in gold).
4. Decidual nest (SHG in gold) + transparent surface rendering of the decidual nest (grey).
5. Transparent surface rendering of the decidual nest (grey).
6. Transparent surface rendering of the decidual nest (grey) + tissue context (THG in blue). THG signal has been cropped around the implantation chamber for clearer visual representation.
7. Transparent surface rendering of decidual nest (grey) + a digital slice of SHG (gold)/THG (blue). Digital slices were used for conceptus and decidual nest measurements in Fig. 7.
8. SHG (gold)/THG (blue) digital slice only.

AM, antimesometrial; E, embryonic day; M, mesometrial.

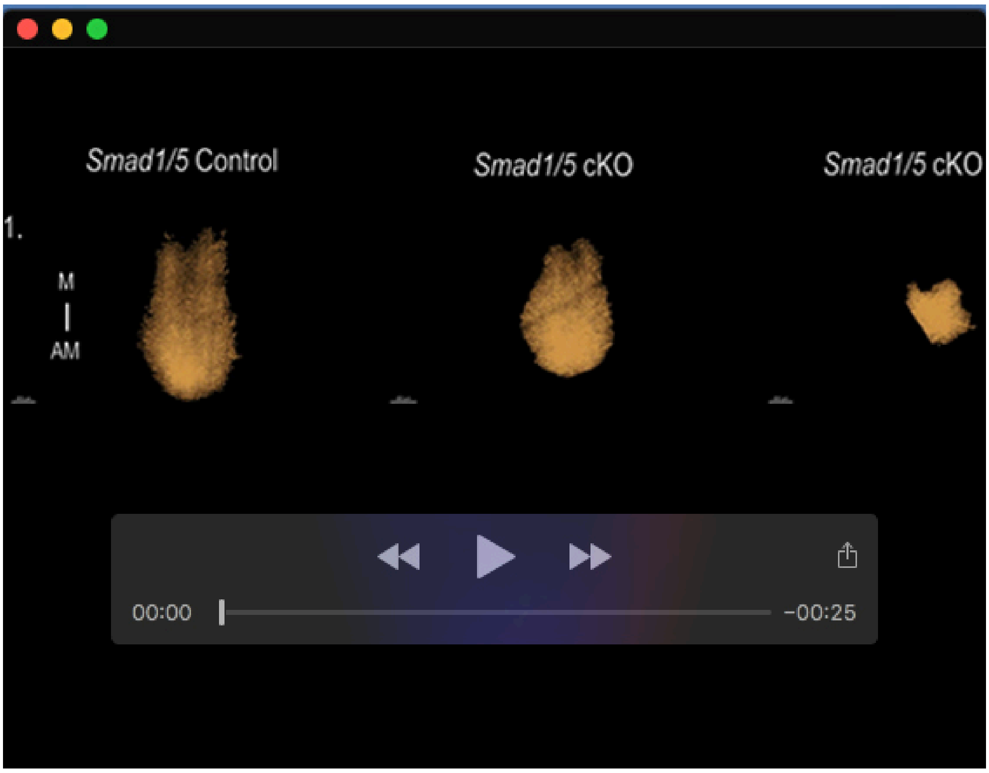

Movie 3. Representative control and *Smad1/5* cKO decidual nests at E5.5. From left to right: *Smad1/5* control decidual nest, *Smad1/5* cKO decidual nest comparable to control in shape, and a poorly developed *Smad1/5* cKO decidual nest. SHG decidual nests are shown in gold and decidual nest transparent surface renderings are in grey. Scale bars for all are 200 µm. The video shows the following progression:

1. Decidual nest (SHG in gold).
2. Decidual nest (SHG in gold) + transparent surface rendering of the decidual nest (grey).
3. Transparent surface rendering of the decidual nest (grey).

AM, antimesometrial; E, embryonic day; M, mesometrial.

Development • Supplementary information

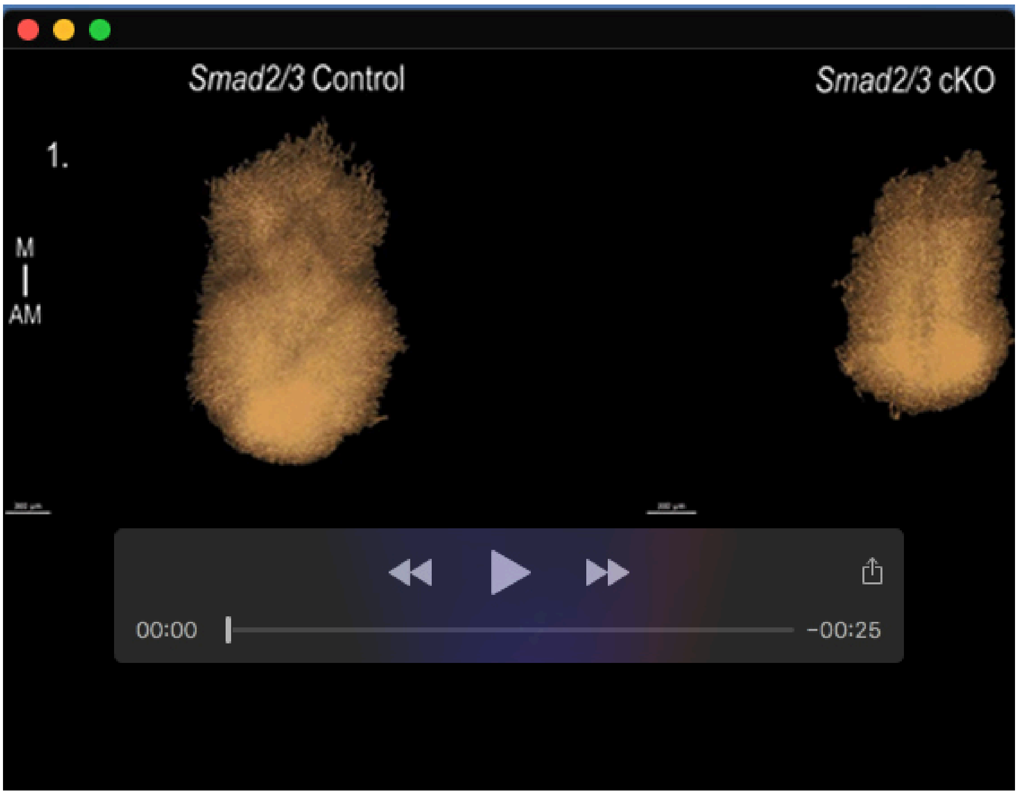

Movie 4. Representative control and *Smad2/3* cKO decidual nests at E6.5. From left to right: *Smad2/3* control decidual nest and *Smad2/3* cKO decidual nest. SHG decidual nests are shown in gold and decidual nest transparent surface renderings are in grey. Scale bars are 300 µm. The video shows the following progression:

1. Decidual nest (SHG in gold).
2. Decidual nest (SHG in gold) + transparent surface rendering of the decidual nest (grey).
3. Transparent surface rendering of the decidual nest (grey).

AM, antimesometrial; E, embryonic day; M, mesometrial.

Development • Supplementary information

Supplementary Materials and Methods

Step-by-step protocol for 3DMOUSEneST

Solutions, materials, and equipment

- 4% paraformaldehyde (PFA) or 10% neutral buffered formalin
    - For 4% PFA, weigh 2 g paraformaldehyde, add 50 mL 1× phosphate buffered saline (PBS), and incubate the solution on a 65°C water bath for approximately 2 h until the PFA is fully dissolved. Store at -20°C and thaw right before use.
- 1×PBS
    - For a 10× stock solution, mix 2.1 g of KH_2PO_4, 90 g of NaCl, and 4.8 g of $Na_2HPO_4 \times 2\ H_2O$ in 1 liter of distilled water. Adjust pH to 7.4 and autoclave. Store at RT.
- Ethanol (EtOH) dilutions
    - 30%, 50%, and 70% EtOH diluted with 1×PBS
- 2:1:3 EtOH: DMSO: H_2O_2 solution
    - Prepare from absolute ethanol, DMSO (cell culture grade) and 30% H_2O_2 stock solution freshly before use
- Sca*le*CUBIC-1 (abbreviated here as CUBIC-1)
    - Susaki et al., 2014, Cell 157(3):726–39
    - Mix 25wt% urea and 25wt% Quadrol (N,N,N'N'-tetrakis(2-hydroxypropyl) ethylenediamine; Sigma Aldrich) while gently heating the mixture (no boiling), and then add 15wt% Triton X-100. Store at RT.
- Mouse tissue collection tools and appropriate infrastructure for mouse work
- Eppendorf tubes of preferred volume
- Tube rotator
- Scalpel or box cutter
- Blunt tweezers
- Blu Tack® (Bostik)
- Pasteur and automate pipettes
- Objective slides
- 24×32 mm #1 coverslips (Menzel-Gläser or Marienfeld)
- 22×22 mm #1 coverslips (Menzel-Gläser)
- Multiphoton microscope
    - upright Nikon A1R MP+ multiphoton microscope with a tunable femtosecond laser (Coherent Chameleon Discovery) and NIS-Elements C-ER acquisition software, or equivalent
- Image analysis software
    - Imaris (Bitplane) or equivalent

Sample collection

Handle the samples in 0.5 mL (single implantation sites) or in 1.5–2 mL (whole uterine horns) Eppendorf tubes until scanning. Use enough solutions to accordingly cover the samples so that they stay well covered in rotation during all sample processing steps.

1. Collect E4.5–E6.5 mouse implantation sites following national and international laboratory animal handling legislation and institutional guidelines.
2. Fix samples (individual implantation sites or whole uterine horns) in 4% PFA at +4°C or in 10% neutral buffered formalin at room temperature (RT) for overnight (o/n).
3. Wash twice for 30 min in 1×PBS, rotating at RT

Development • Supplementary information

4. Dehydrate samples stepwise to 70% EtOH
   - Move samples to 30% EtOH, 50% ETOH, and then 70% EtOH, rotating for a minimum of 15 min at RT for each step.

Samples can be stored at this stage in 70% EtOH at +4°C for months (up to years) or can be taken for quenching and clearing immediately.

Sample quenching and optical clearing

5. Quench samples by incubating in 2:1:3 EtOH: DMSO: H_2O_2 rotating at RT for o/n.
   - Quenching is performed to remove autofluorescence and tissue coloration.
6. Wash samples in 70% EtOH rotating at RT for a minimum of 2 × 30 min
7. Rehydrate samples stepwise to 1×PBS, rotating at RT
   a. 50% EtOH for 30 min
   b. 30% EtOH for 30 min
   c. 1×PBS for 30 min
   d. 1×PBS for 30 min–o/n
8. Clear the samples in CUBIC-1, rotating at RT for 4–5 days, changing the CUBIC-1 daily to fresh.
   - Clearing will take about 4–5 days, depending on sample size. The samples will be transparent when ready.

After samples are cleared, they can be stored in CUBIC-1 at +4°C for years. If a sample has been stored in CUBIC-1 for a long time and the CUBIC-1 is starting to crystallize, move the sample to fresh CUBIC-1 and leave rotating at +4°C for o/n to dissolve crystals.

Preparing the sample chamber for scanning

9. Move the samples to RT about 1 h before scanning to warm them up (improves scanning quality).
10. Roll out Blu Tack® into a "worm" that is about 3 mm in diameter.
11. Make a loop of Blu Tack® and be sure that the connection point is well sealed but not thinner than the rest of the ring.
    - Depending on whether scanning one implantation site or an entire horn, loop size will be different: one implantation site needs about the circumference of a thin finger, while one uterine horn is about 2 cm in length
12. Place the Blu Tack® loop onto the 24×32mm coverslip.
    - Note: This protocol provides details for a sample chamber intended to be imaged in both epi and trans directions, so coverslips are used both as the base and the lid to reduce chamber thickness. In cases when imaging is done only from the epi direction, objective glass slides can also be used for the chamber base.
13. Use a clean objective slide to press the Blu Tack® onto the coverslip. Check the underside of the coverslip to see that the Blu Tack® is well attached.
14. With a scalpel or box cutter, gently pry the objective glass from the Blu Tack® without lifting the Blu Tack® from the coverslip base.
15. Place the implantation site into the Blu Tack® ring with tweezers.
    - If the implantation site touches the top of the Blu Tack® ring, gently wipe off any CUBIC-1 left on the top of the Blu Tack® before covering it with a coverslip as moisture can prevent its attachment.
    - To orient the implantation site, lay it on its side so that the lumen is running parallel to the table (rather than standing up on the cut end of the implantation site).

Development • Supplementary information

16. Add a small amount of CUBIC-1 to the Blu Tack® ring containing the implantation site using a Pasteur pipette.
    - Note that this is a very small amount: do not cover the sample with CUBIC-1. For a single implantation site, a drop or two with the Pasteur pipette is usually sufficient.
17. Place a 22×22 mm coverslip on the Blu Tack® ring and use a clean objective glass to gently press the top coverslip in place.
    - Avoid many bubbles! One bubble that will not touch the sample can still be managed. Gently tilt the sample chamber to move the bubble out of the way of the sample.
    - If CUBIC-1 leaks out from under Blu Tack® (either on the top or the bottom), the imaging chamber needs to be remade from the beginning. Otherwise, the sample can dry during scanning and the Blu Tack® will not stick to the coverslip once it is wet.

SHG and THG scanning

We have used an upright Nikon A1R MP+ multiphoton microscope equipped with a tunable femtosecond laser (Coherent Chameleon Discovery) and NIS-Elements C-ER acquisition software. However, any multiphoton microscope with an appropriate setup for SHG and THG imaging can be used.

18. Position the sample chamber in the microscope using water immersion with a 16× (NA 0.8) objective lens and condenser for whole decidual nest scanning.
    - For decidual nest imaging, this is adequate resolution. If more detailed close-up images are desired, the objective can be selected accordingly.
19. Set excitation wavelength for SHG and THG to 1100 nm, where the SHG signal is expected at half the excitation wavelength and the THG signal is expected at 1/3 the excitation wavelength.
    - In our setup, described in Materials and Methods, we observed that trans SHG and epi THG signals gave the best contrast, where trans SHG emission is collected between 495 and 560 nm and epi THG is collected under 458 nm short pass. If desired, also collect SHG emission in epi and THG emission in trans directions with suitable wavelengths.
    - Of note, using a narrow emission detection window (*e.g.*, 10 nm) surrounding 1/3 and 1/2 of the excitation wavelength allows for more specific THG and SHG signal detection, respectively. However, the wider detection range for THG (collecting below 458 nm short pass) provides better signal for analysis of THG scans.
    - If applicable, fluorescent signals stemming from genetic reporter markers or immunostaining fluorophores can be imaged simultaneously.
20. Locate the decidual nest by navigating through the sample for the fibril accumulation using SHG.
21. After finding the decidual nest, use SHG/THG to navigate through the nest to the center, where the embryo outline will be visible. In the ideal orientation, the decidual nest will surround the embryo in a “U” shape.
22. At the center of the nest, where the embryo outline is visible, a large multi-tile overview image can be acquired to visualize the nest in relation to the myometrium and uterine lumen. Additionally, as this will be the largest section of the nest, it can be used to determine the desired number of tiles needed for 3D imaging to capture the entire decidual nest. This will vary based on implantation site age and size.
23. When using tile imaging, select stitching to be in use and set overlap to 10–30%. Test different overlap settings to find such settings that do not cause stitching artefacts.
24. After selecting the number of tiles, set the range of the scan.
25. Set the desired step size, usually 4–5 µm.
26. Set the desired resolution, usually 512×512 for rough scans and 1024×1024 for decidual nest imaging. *x*×*y* pixel sizes are 1.59 µm or 0.79 µm, respectively.

27. Set laser power with gradually increasing intensity to maintain even brightness as the z-direction depth increases.
28. Scan the sample. Scanning time is highly dependent on the resolution, number of steps, size of the sample, and number of tiles.
    - A general scanning time for an E5.5 sample (4 µm steps, 1000 µm range, 1024×1024 resolution, pixel size 0.79 µm, 3×2 tiles) is 2 h.
29. After scanning, disassemble the sample chamber and return samples to CUBIC-1 for storage at +4°C.

Image analyses

We have used Imaris software (Bitplane, version 9.8.0) for image analyses. However, any software with appropriate features and file type compatibility can be used. All Nikon multiphoton files were converted from .nd2 files to Imaris .ims files using the Imaris File Converter.

Decidual nest volume measurements

30. Open Imaris files. Multiphoton scan images are reconstructed into 3D automatically when viewed using "Volume" mode. Decidual nest volume is measured using the "Surfaces" feature.
31. Select "Create algorithm" in the "Surface" feature to generate a surface for the decidual nest.
32. Select trans direction SHG channel as "Source Channel".
33. Set surface detail to 15 µm, which is optimized to cover the border of SHG decidual nest signal.
34. Set manually the threshold for recognizing decidual nest border using the slicer view to best recognize nest and exclude myometrium.
35. Delete myometrium surface, leaving only decidual nest for measurements.
36. In cases where the myometrium and decidual nest touch in the surface rendering, use the editing tool "Cut Surface" to separate myometrium from the decidual nest.
37. To measure the volume, use "Statistics" feature, select the nest surface, and choose "Volume".

Conceptus and decidual nest dimension measurements

38. To measure conceptus growth in relation to the decidual nest, use trans SHG and epi THG channels, viewing the decidual nest and conceptus in 3D "Volume" mode.
39. Orient the decidual nest so that its top (the mesometrial side) is towards the top of the screen and the bottom (the antimesometrial side) is towards the bottom of the screen.
40. Use the "Oblique" plane tool to find the digital slice in the 3D "Volume" that shows the widest, central part of the conceptus.
41. Once this digital slice is selected, deselect "Volume."
42. Make desired measurements using "Measurement points" with only the "Oblique" plane activated. Select the option "Intersect" with "Surface of Object".

Timeline estimation for 3DMOUSEneST

- Sample collection, fixing, and washing (steps 1–4)
    - active (hands-on): approx. 2–6 h, highly depending on the number of samples collected and processed
    - passive (waiting): overnight
- Quenching and rehydration (steps 5–7)
    - active: approx. 2 h, with short waiting gaps in between washes
    - passive: 1–2 days depending on rehydration time
- Optical clearing (step 8)

Development • Supplementary information

  - active: approx. 10 min/sample
  - passive: 4–5 days
- Chamber preparation (steps 9–17)
  - active: approx. 15 min/sample
- Scanning (steps 18–29)
  - active: approx. 30 min/sample
  - passive: approx. 2 h/sample
- Measuring decidual nest volume (steps 30–37)
  - active: approx. 10–30 min/sample
- Optional: Measuring conceptus and decidual nest dimensions using digital slices (steps 38–42)
  - active: approx. 10–30 min/sample

See Fig. 4 for a graphical representation of the workflow timeline.

Development • Supplementary information
