# Supplementary material for: 3DMOUSEneST: a volumetric label-free imaging method evaluating embryo–uterine interaction and decidualization efficacy
Source: Development. 2024 Aug 29;151(16):dev202938. doi: 10.1242/dev.202938 (PMC11385321; doi:10.1242/dev.202938)
Supplement: Supplementary file 1 [file develop-151-202938_review_history.pdf]

3DMOUSEneST: a volumetric, label-free imaging method evaluating embryo-maternal interaction and decidualization efficacy

Audrey Savolainen, Emmi Kapiainen, Veli-Pekka Ronkainen, Valerio Izzi, Martin M Matzuk, Diana Monsivais and Renata Prunskaite-Hyyrylainen
DOI: 10.1242/dev.202938

Editor: Liz Robertson

Review timeline

Original submission:	13 December 2023
Editorial decision:	22 January 2024
Resubmission:	16 April 2024
Editorial decision:	24 May 2024
First revision received:	4 July 2024
Accepted:	10 July 2024

Original submission

First decision letter

MS ID#: DEVELOP/2023/202603
MS TITLE: 3DMOUSEneST: a volumetric, label-free imaging method evaluating embryo-maternal interaction and implantation efficacy

AUTHORS: Renata Prunskaite-Hyyrylainen, Audrey Savolainen, Emmi Kapiainen, Veli-Pekka Ronkainen, Valerio Izzi, Martin M. Matzuk, and Diana Monsivais,
ARTICLE TYPE: Research Article

I have now received all the referees reports on the above manuscript, and have reached a decision. The referees' comments are appended below, or you can access them online: please go to BenchPress and click on the 'Manuscripts with Decisions' queue in the Author Area.

As you will see from their reports, the referees recognise the potential of your work, but they also raise significant concerns about it. Given the nature of these concerns, I am afraid I have little choice other than to reject the paper at this stage.

However, having evaluated the paper, I do recognise the potential importance of this work. I would therefore be prepared to consider as a new submission an extension of this study that contains new experiments, data and discussions and that address fully the major concerns of the referees. The work required goes beyond a standard revision of the paper. Please bear in mind that the referees (who may be different from the present reviewers) will assess the novelty of your work in the context of all previous publications, including those published between now and the time of resubmission.

Reviewer 1

Advance summary and potential significance to field

The authors in the current manuscript imaged implantation sites 3 dimensionally using second-harmonic generation (SHG) to detect fibrillar collagens and third-harmonic generation (THG) to

© 2024. Published by The Company of Biologists under the terms of the Creative Commons Attribution License (https://creativecommons.org/licenses/by/4.0/).

detect water-lipid/protein interfaces. This method can review the collagens formed in the secondary decidual zone in early pregnancy. This study also compared the natural and artificial decidua, showing collagen fibrils are more organized in natural decidua. Using SHG/THG signals, the authors tried to quantify embryo/decidual bed sizes, and used the measurement to predict implantation quality. The method has its advantages as compared to 3D imaging using antibodies. The current method doesn't need antibody staining. However, an obvious limitation is the presence of fibrillar collagens, so the authors focused on E4.5 to E6.5 implantation chambers. The methods should be introduced to the reproductive society, so that more groups could finely define the relationship between the signals and actual embryo/decidua.

Comments for the author

My main concern is that some statements in the manuscript are not supported by solid data. My concerns are narrated below:

The authors stated "To our knowledge, this is the first report of the 3D organization of decidual collagen in mice." The authors need to show collagen stainings side by side in terms to make this claim.

The authors claimed that "We noticed a strong THG signal coinciding with the conceptus, where the embryo was observed as a dark "hollow" encapsulated by THG signal likely coming from the extraembryonic tissues." It's hard to judge what the "hollow" represents comparing H&E staining and THG signals. It seems it could represent blastocele (the cavity of the blastocyst) in 1E, a mixture of the embryo proper and empty spaces in 1F, and the embryo proper in 1G. In addition, the THG signals are much more than extraembryonic tissues comparing 1G and 1C'. To evaluate what the THG signals stand for, the authors need to do more staining examinations to carefully compare the signals.

The SHG signals in 1A are little, but the signals in 1E are clear. Why do they show the difference?

What is the purpose of showing trichrome staining in figure 1? Comparing 1B and 1B', I don't see any similarity.

E5.5 BMP2 in situ signals usually show bigger domains than the signals of SHG. I think SHG signals cover partial decidual zones. This may raise a new decidual concept using collagen expressions. However, the authors should describe this more carefully. I don't think all SHG signals should be explained by old/established concepts.

Figure 1D: SHG signals are in gold; THG signals are in blue; what are the grey signals in the 3D reconstruction picture?

In figure 3, the tomato+ embryo from E6.5 does not look typical. A typical E6.5 embryo looks like the one shown in figure 1C'. Embryos on E6.5 usually show a solid mass with minimal cavities.

The measurement of embryos in figure 4 focuses on the "hollow" hole, which is different from the tomato signals in figure 3. Tomato+ embryonic cells are not located in the hollow hole of THG signals.

One suggestion: since this is a paper on a new method, it would be helpful for the reader to appreciate the new method if the authors could move some method descriptions in the result section. A little comparison of the current method with old 3D methods would also be helpful. I hope more groups can join and push the limit of the current method to broader gestational times or other organs.

Reviewer 2

Advance summary and potential significance to field

© 2024. Published by The Company of Biologists under the terms of the Creative Commons Attribution License (https://creativecommons.org/licenses/by/4.0/).

In this manuscript authors claim to establish a technique that provides volumetric, label-free imaging to evaluate embryo-maternal interaction and implantation efficiency. The authors first use established second harmonic generation techniques to the pregnant uterus and identify collagen networks in the newly formed decidua. They then claim to use third harmonic generation to identify the embryo. Although the premise is exciting there is a lack of rigor and the data provided do not support identification of the embryo using THG signal. The methodology also does not capture embryo-uterine interactions as suggested by the title.

Comments for the author

Major comments:

-The authors claim that the methodology will allow them to evaluate embryo-uterine interactions. However, the label free imaging is only detecting decidualizing stroma. It is not detecting the luminal epithelium or the embryo which is where the interactions are occurring at E4.5.

-Figure 3 shows the use of labeled tomato embryos and how the tomato signal overlaps with THG signal. The authors say that the tomato label corresponds to the embryo. What part of the embryo is the tomato label? The authors at the same time say that the embryo is the dark hollow. So, it is unclear that the tomato label is indicative of the embryo. Why is there tomato label outside the implantation chamber? In supplementary video 2 the tomato label spreads throughout the mesometrial pole of the implantation chamber suggesting that tomato label as reported in figure 2 and supplementary video 2 does not correspond to the embryo. Since tomato is only expressed by the embryo and not the uterus, this reviewer is unsure if the tomato label is even specific. In figure 5 the THG signal clearly corresponds to the uterine lumen as labeled by the authors. To determine if the tomato signal is the embryo authors need to section the decidua and colocalize tomato signal with the THG signal and a marker specific to the embryo. Further similar to Figure 5 that shows multiple implantation sites, if tomato is truly the embryo, then a similar view to figure 5 should be shown with the tomato label and the THG label as separate panels. Overlapping the THG and the tomato signal makes it harder to see the expanse of the tomato label.

-Figure 6 is where the authors claim that this methodology can be used for predictions of pregnancy. However, it is impossible to test this idea. In models of delayed implantation there may be reduced number of nests that will expand once pregnancy catches up. In the absence of molecular markers, the methodology can only tell at a given time point how much decidua has accumulated using collagen signal as proxy for decidualization.

-The organization of primary and secondary decidual zones as described by the authors is not in line with the spatial locations of primary and secondary decidual zones described in the literature. To establish their claims, authors need to use known molecular markers for the different zones.

-The authors coin the term decidual nests. However, it has been long known that collagens and ECM accumulates around the newly formed decidua.

-Authors claim that this methodology will indicate implantation efficiency. Implantation occurs between E4.0-E4.5 and decidual nests by name are indicative of decidualization. So, the method can predict if decidualization is happening and how much decidualization is happening (not implantation efficacy).

-The authors claim that this methodology is non-destructive. However, since the tissue is fixed and cleared there method is comparable to other techniques where samples are fixed and cleared after immunolabeling.

-The authors claim that using this methodology will reduce usage of mice. However, since this is not live imaging animals will have to be dissected at different time points to acquire data as with other technologies that use fixed tissue and reconstruct 3D structures. It is possible to use previously cleared tissue for histology but the quality of histology for samples that are pre-cleared is not shown. To support their claim that material can be reused, the authors should perform SHG analysis on 3D cleared tissue and then provide 2D histology images to back the claim that the material can be used for tissue sectioning once SHG is done.

© 2024. Published by The Company of Biologists under the terms of the Creative Commons Attribution License (https://creativecommons.org/licenses/by/4.0/).

-Figure 2 – Authors claim that in artificial decidualization fibrils are distributed sporadically compared to natural decidualization where collagen fibers have a more orderly organization. There is no quantification to back this data up. How many mice and how many decidual sites were evaluated? Based on the methods the samples from natural matings and those for artificial decidualization were fixed in different fixatives. Why were the samples not processed the same way? The organization of collagen fibers could easily be different because the tissue was fixed in different fixatives for the two samples being compared.

-In figure 6 not all genotypes are shown in panel K for all the time points and it is unclear why some genotypes were omitted.

-Do collagen fibrils in smadKOs organize normally or are they sporadic as the authors claim for artificial decidualization?

-Authors claim that the entire process is faster than other 3D protocols. What is the timeline for the entire protocol from dissection to imaging? What is the resolution of their method compared to published 3D methods.

-A big section in the introduction is duplicated.

-Line 51 "Presently, many studies on early implantation sites still rely on traditional, labor-intensive two-dimensional (2D) microtome sectioning of paraffin-embedded tissue yielding only a few tissue sections for future analysis (Arora et al., 2016; Pang et al., 2014)." Arora et al 2016 is a study that describes 3D imaging of the entire uterine horn with immunolabeling and tissue clearing. It is not a 2D study.

First revision

Author's response to the reviewer's comments

Reviewer 1 Advance Summary and Potential Significance to Field:
The authors in the current manuscript imaged implantation sites 3 dimensionally using second-harmonic generation (SHG) to detect fibrillar collagens and third-harmonic generation (THG) to detect water-lipid/protein interfaces. This method can review the collagens formed in the secondary decidual zone in early pregnancy. This study also compared the natural and artificial decidua, showing collagen fibrils are more organized in natural decidua. Using SHG/THG signals, the authors tried to quantify embryo/decidual bed sizes, and used the measurement to predict implantation quality. The method has its advantages as compared to 3D imaging using antibodies. The current method doesn't need antibody staining. However, an obvious limitation is the presence of fibrillar collagens, so the authors focused on E4.5 to E6.5 implantation chambers. The methods should be introduced to the reproductive society, so that more groups could finely define the relationship between the signals and actual embryo/decidua.

Reviewer 1 Comments for the Author:
My main concern is that some statements in the manuscript are not supported by solid data. My concerns are narrated below: The authors stated 'To our knowledge, this is the first report of the 3D organization of decidual collagen in mice.' The authors need to show collagen stainings side by side in terms to make this claim.

➔ We thank the reviewer for helping us realize that the reasoning for associating SHG with fibrillar collagen was unclear in the previous manuscript version. SHG has been established to be an intrinsic property highly specific for fibrillar collagen-only myosin and microtubules exhibit similar properties, and among these, fibrillar collagen is the most abundant structure in tissues (for references, see, e.g., Strupler et al. 2007 Opt Express. 15(7):4054-65; Aghigh et al. 2023 Biophys Rev. 15(1):43-70). We have now added more information on SHG (and THG) imaging in the

© 2024. Published by The Company of Biologists under the terms of the Creative Commons Attribution License (https://creativecommons.org/licenses/by/4.0/).

Introduction chapter with references (lines 89-100). In the previous version of the manuscript, the Masson's trichrome stainings were intended to verify that SHG signal stems from collagen. To provide more convincing evidence, we now show new Picrosirius red stainings. Apart from collagen IV, Picrosirius red staining is highly specific for fibrillar collagens when imaged with standard light microscope, and once polarized light is used to image Picrosirius red staining only the staining stemming from fibrillar collagen is depicted (for references, see, e.g., Junqueira et al. 1979 Histochem J 11:447-455; Montes & Junqueira LC, 1991, Mem Inst Oswaldo Cruz 86 Suppl 3:1-11; Lattouf et al. 2014 J Histochem Cytochem. 62(10):751-8). We first imaged implantation site sections for SHG (new Fig. 1A-C') and then stained the same tissue sections with Picrosirius red, which was imaged both in brightfield and with polarized light (new Fig. 1D-I) to specifically verify that the SHG signal indeed stems from fibrillar collagen in the decidua. Of note, we decided to leave the Masson trichrome staining data as a validation for SHG and decidual cell colocalization determined by not only collagen but also the distinct decidual cell morphology (large multinucleated cells), which the Masson's trichrome staining well visualizes. We moved the data to the new Fig. 3 and replaced the previous images with higher resolution images for more convincing validation (see also point below on decidual zone determination).

The authors claimed that 'We noticed a strong THG signal coinciding with the conceptus, where the embryo was observed as a dark 'hollow' encapsulated by THG signal likely coming from the extraembryonic tissues.' It's hard to judge what the 'hollow' represents comparing H&E staining and THG signals. It seems it could represent blastocele (the cavity of the blastocyst) in 1E, a mixture of the embryo proper and empty spaces in 1F, and the embryo proper in 1G. In addition, the THG signals are much more than extraembryonic tissues comparing 1G and 1C'. To evaluate what the THG signals stand for, the authors need to do more staining examinations to carefully compare the signals.

➔ We thank the reviewer for the insightful comment. To better clarify the structures generating THG signals we have now performed immunostaining with anti-laminin antibody depicting the conceptus-enveloping Reichert's membrane and overlayed it with the THG signal of the same sample. This demonstrated that the brightest THG signal is located just outside the conceptus, encapsulating the conceptus along the Reichert's membrane (new Fig. 6A-C). Furthermore, we quantified the emitted THG signal intensity ratio of surrounding uterine tissue to the conceptus to demonstrate a significant difference in signal intensity between the maternal and embryonic tissues (new Fig. 6D). Compared to the surrounding tissues, the conceptus showed weaker THG signals and is therefore defined as the dark 'hollow'. We have now corrected our terminology from 'embryo' to 'conceptus' in relevant places throughout the manuscript, e.g., in new Fig. 7 for the conceptus measurements.

The SHG signals in 1A are little, but the signals in 1E are clear. Why do they show the difference?

➔ They are different samples that have also been differentially processed and imaged. Previous Fig. 1A (a similar image is now presented in new Fig. 1A and the previous 1A is now shown in Fig. 3A) is a 5 µm thick section of paraffin-embedded, not chemically cleared tissue, while previous Fig. 1E, now Fig. 4C', is a digital slice derived from a chemically cleared whole implantation site scanned in 3D. The collagen accumulation around the newly implanted embryo at E4.5 is indeed low. The E4.5 3D implantation sites are presented with a zoomed-in view (note scale bars in the images). However, the major reason for seeing seemingly more enhanced collagen accumulation at E4.5 in 3D scanned specimens as compared to the 2D sections is associated with the thicker digital sections in 3D view data depicting collagen accumulation through the implantation site. Additionally, at E4.5, even the slightest changes in embryonic age determination can significantly affect the extent of detected collagen accumulation. By moving SHG paraffin section images into their own figure (new Fig. 1A-C) and making the new Fig. 4 solely focusing on the 3DMOUSEneST we have now aimed to make the difference clearer to the reader.

What is the purpose of showing trichrome staining in figure 1? Comparing 1B and 1B', I don't see any similarity.

➔ Masson's trichrome staining depicts collagen and was meant to demonstrate SHG signal and collagen colocalization. In the current manuscript we have expanded this study and added Picrosirius red staining. Please also see the answer above on SHG correlation with collagen

© 2024. Published by The Company of Biologists under the terms of the Creative Commons Attribution License (https://creativecommons.org/licenses/by/4.0/).

staining.

E5.5 BMP2 in situ signals usually show bigger domains than the signals of SHG. I think SHG signals cover partial decidual zones. This may raise a new decidual concept using collagen expressions. However, the authors should describe this more carefully. I don't think all SHG signals should be explained by old/established concepts.

➔ *Bmp2*, and another common decidual marker *Wnt4*, are, to the best of our knowledge, studied in decidua only using mRNA expression analysis. In situ hybridization, i.e., gene expression, precedes protein translation. Fibrillar collagen is protein deposited by the decidualized cells in response to decidualization, and it is thus detected around already truly decidualized cells, and not yet around the cells just initiating differentiation to become decidual cells. For this reason, *Bmp2* in situ hybridization may well show a slightly broader decidualization ring in comparison to fibrillar collagen (SHG signal). We agree that SHG signals may present an alternative way for determining decidual zones given how the range of markers and definitions, especially for the secondary decidual zone (SDZ), has not been perfectly unambiguous (see, e.g., Paria et al. 2001 PNAS 98(3):1047-52; Yuan et al. 2019 Nat Commun. 10(1):5425; Favaro et al. 2014, The Guide to Investigation of Mouse Pregnancy, p. 125-142; Mori et al. 2016 Semin Immunopathol. 38(6):635-649; Maurya et al. 2021 Front Cell Dev Biol 9:640907; Panja & Paria 2021 Adv Anat Embryol Cell Biol. 234: 205-221). We present immunostaining with an established primary decidual zone (PDZ) marker PTGS2 (COX-2) in the current Fig. 2 to demonstrate how the SHG signal does not originate from the PDZ at E4.5-E6.5. We also show Masson's trichrome stainings (new Fig. 3) to indicate that the SHG signal well corresponds to the area with decidualized cells distinguished by their enlarged, multinuclear morphology. Furthermore, in the new Fig. 1, we now more carefully present the matching of SHG signal with fibrillar collagen using Picrosirius red staining. Thus, we state that the SHG signal is not stemming from the PDZ but rather from the more outer decidual zone, which usually is referred to as the SDZ, although, as stated above, the exact SDZ definition seems to vary. We envision that the 3DMOUSEneST method can be used in the future to address these discrepancies to establish intrinsic clues for precise SDZ definition as it depicts an intrinsic feature stemming from the tissue itself avoiding biases related to in situ or antibody staining.

Figure 1D: SHG signals are in gold; THG signals are in blue; what are the grey signals in the 3D reconstruction picture?

➔ The grey color is a transparent surface rendering generated by the analysis software based on 3D SHG signal reconstruction and it is used to additionally see through the decidual nest structure and to provide better contrast in comparison to golden, opaque SHG surface renderings. Especially the Supplementary Movie 1 shows the SHG signal (gold), the THG signal (blue), and the grey, transparent surface (surface of SHG signal) in relation to one another, specifically demonstrating how the grey, transparent surface fits over the SHG signal. The grey color was chosen so that the SHG signal (gold) and the surface rendering (grey) can be shown at the same time and be visually distinct (Fig. 4A and Supplementary Movies 1-3). We acknowledge that the differences between the image types included in the manuscript were not adequately explained. We have now better clarified them in the Results section (lines 164-174) and in Fig. 4 and its corresponding figure legend.

In figure 3, the tomato+ embryo from E6.5 does not look typical. A typical E6.5 embryo looks like the one shown in figure 1C'. Embryos on E6.5 usually show a solid mass with minimal cavities.

➔ The previous Fig. 3 was a representative sample among the *mTmG* samples we had, showing brighter tdTomato signal around the embryo than on the embryo/conceptus itself. However, we removed the *mTmG* data from the manuscript and replaced it with a more comprehensive analysis of the THG signal in relation to the conceptus using laminin immunostaining to visualize the Reichert's membrane (new Fig. 6). This shows how brighter THG signal envelops the conceptus, which is dimmer in terms of THG and therefore appears as a dark 'hollow' (please also see more detailed, related answer above).

The measurement of embryos in figure 4 focuses on the 'hollow' hole, which is different from the tomato signals in figure 3. Tomato+ embryonic cells are not located in the hollow hole of THG

© 2024. Published by The Company of Biologists under the terms of the Creative Commons Attribution License (https://creativecommons.org/licenses/by/4.0/).

signals.
➔ As mentioned right above, we have now removed *mTmG* data and show more convincingly in the new Fig. 6 how brighter THG signal envelops the conceptus, which, outlined by the laminin-positive Reichert's membrane, is dimmer in terms of THG and therefore appears as a dark 'hollow' especially in the chemically cleared samples from which the measurements are taken. THG signal thus provides an inverse way to detect the conceptus. In new Fig. 7, measurements of the 'hollow' are now accordingly called measurements of the conceptus.

One suggestion: since this is a paper on a new method, it would be helpful for the reader to appreciate the new method if the authors could move some method descriptions in the result section.
➔ We thank the reviewer for this important note and have now added more description of the complete 3DMOUSEneST workflow and the image types into the Results text (lines 158-175) and in new Fig. 4 and its legend to help the reader better understand the obtained results. We now also provide a supplementary step-by-step protocol of the 3DMOUSEneST (new Supplementary Materials and Methods) to facilitate its application in other research laboratories.

A little comparison of the current method with old 3D methods would also be helpful. I hope more groups can join and push the limit of the current method to broader gestational times or other organs.
➔ Indeed, we show a novel view on the conceptus, decidua, and early implantation site. We provide an array of experimental data and bridge it with an established view to facilitate knowhow expansion. We do discuss the pros and cons of other currently used implantation site 3D imaging techniques and also add in comparison with the 3DMOUSEneST intertwined both in the Introduction and in the Discussion chapters to help the readers to define the applicability and power of the proposed methodology (e.g., lines 53-71; 309-319). A more detailed comparison could take its place after 3DMOUSEneST has solidified its place in the field. Like the reviewer, we wish that more groups will join us in testing and developing this methodology, enabling its broader applicability and scientific advancement.

Reviewer 2 Advance Summary and Potential Significance to Field:
In this manuscript authors claim to establish a technique that provides volumetric, label-free imaging to evaluate embryo-maternal interaction and implantation efficiency. The authors first use established second harmonic generation techniques to the pregnant uterus and identify collagen networks in the newly formed decidua. They then claim to use third harmonic generation to identify the embryo. Although the premise is exciting there is a lack of rigor and the data provided do not support identification of the embryo using THG signal. The methodology also does not capture embryo-uterine interactions as suggested by the title.

Reviewer 2 Comments for the Author:
Major comments:

The authors claim that the methodology will allow them to evaluate embryo-uterine interactions. However, the label free imaging is only detecting decidualizing stroma. It is not detecting the luminal epithelium or the embryo which is where the interactions are occurring at E4.5.
➔ We thank the reviewer for the perceptive comment and appreciate that there may be variable point-of-views on this matter. However, it often goes without adequate recognition that beyond being a passive macromolecular meshwork for cells to reside in, the ECM molecules interact with other ECM components, with cell surface receptors, and with growth factors to mediate crucial cellular processes, and ECM composition varies depending on tissue compartments or time points (roles of decidual ECM are reviewed in Favaro et al. 2014, The Guide to Investigation of Mouse Pregnancy, p. 125-142). The decidua has several important roles in early embryo development, including protection of the embryo from immune cell attacks and providing nutritional support (Mori et al. 2016 Semin Immunopathol. 38(6):635-649). Even though we do not detect the decidual cells as such using 3DMOUSEneST, we can directly detect the decidual fibrillar collagen and indirectly embryonical structures. While one view is to consider only the most direct luminal epithelium-embryo interactions, the dynamic decidual ECM is essentially interacting with the

© 2024. Published by The Company of Biologists under the terms of the Creative Commons Attribution License (https://creativecommons.org/licenses/by/4.0/).

decidual cells, which in turn interact towards the embryo, and thus we argue our method helps in assessing embryo-uterine interactions when they are considered from this wider perspective. For example, decidualization reaction is initiated as a response to the physical attachment of an embryo despite it never having a direct connection with the endometrial stroma. Additionally, we provide data comparing natural and artificial decidualization which suggests that the presence of an embryo provides a directing effect on the decidual fibrillar collagen, and that the embryo-uterine interaction lasts beyond E4.5. We have now added discussion on the active role of ECM in tissues and how embryo-maternal interactions can be determined to the Discussion chapter (lines 335-340).

Figure 3 shows the use of labeled tomato embryos and how the tomato signal overlaps with THG signal. The authors say that the tomato label corresponds to the embryo. What part of the embryo is the tomato label? The authors at the same time say that the embryo is the dark hollow. So, it is unclear that the tomato label is indicative of the embryo. Why is there tomato label outside the implantation chamber? In supplementary video 2 the tomato label spreads throughout the mesometrial pole of the implantation chamber suggesting that tomato label as reported in figure 2 and supplementary video 2 does not correspond to the embryo. Since tomato is only expressed by the embryo and not the uterus, this reviewer is unsure if the tomato label is even specific. In figure 5 the THG signal clearly corresponds to the uterine lumen as labeled by the authors. To determine if the tomato signal is the embryo authors need to section the decidua and colocalize tomato signal with the THG signal and a marker specific to the embryo. Further similar to Figure 5 that shows multiple implantation sites, if tomato is truly the embryo, then a similar view to figure 5 should be shown with the tomato label and the THG label as separate panels. Overlapping the THG and the tomato signal makes it harder to see the expanse of the tomato label.

➔ We thank the reviewer for these important notes that made us re-evaluate our data and provide new data to strengthen our claims. We recognize that the *mTmG* data was unclear. Given this, we have now removed the *mTmG* data from the manuscript. Instead, to clarify the origin of the THG signal, we have used anti-laminin antibody to immunostain the conceptus-encapsulating Reichert's membrane and overlayed that with the THG signal image. This demonstrated that the brightest THG signal surrounds the conceptus along the Reichert's membrane whereas the conceptus itself appears dimmer (new Fig. 6). We hope that this new data allows clearer understanding of the THG signal. THG signal in general stems from various structural interfaces and usually is not highly specific to any particular structure (Weigelin et al. 2016 J Cell Sci. 129(2):245-55). In implantation sites, we can thus use THG to provide context for recognizing the conceptus. Accordingly, we have now corrected our terminology from 'embryo' to 'conceptus' in relevant places throughout the manuscript, e.g., in new Fig. 7 for the conceptus measurements.

Figure 6 is where the authors claim that this methodology can be used for predictions of pregnancy. However, it is impossible to test this idea. In models of delayed implantation there may be reduced number of nests that will expand once pregnancy catches up. In the absence of molecular markers, the methodology can only tell at a given time point how much decidua has accumulated using collagen signal as proxy for decidualization.

➔ It is true that we cannot follow through the pregnancy development of the same specimen when using ex vivo methods. In reference to the reduced number of nests, the number of nests will likely not increase as that should be the same or less as the number of very initial implantation sites. If the reviewer is referring to the reduced volume of nests due to delayed implantation, we acknowledge that this is a possible scenario. Our data, shown in Fig. 9K, illustrates the range of normal variability that exists in control samples, which takes into account delayed implantation and growth rate. Our logistic regression model is built using the decidual nest volumes from controls and conditional knockout mouse models with known decidualization defects and defects in sustaining pregnancy, showing 92.3% accuracy, which is high-but not 100%, and we acknowledge it is possible to misassign a decidual nest. Based on the presented evidence and the published phenotypes of the *Smad1/5* and *Smad2/3* cKO implantation sites, we, however, have high confidence in suggesting that fibrillar collagen signal can be used as a proxy for decidualization efficacy (current Fig. 9). We agree that this is an indirect readout for pregnancy progression success, but it is still well correlating with the known phenotypes of the presented mouse models. Analysis of decidual nests indicates decidualization efficacy, which can be used as an ex vivo

© 2024. Published by The Company of Biologists under the terms of the Creative Commons Attribution License (https://creativecommons.org/licenses/by/4.0/).

measure to estimate the potential the early pregnancy had to progress further. We have now rephrased the text throughout the manuscript to better indicate that the 'pregnancy prediction' is referring to the 'potential for pregnancy progression.'

The organization of primary and secondary decidual zones as described by the authors is not in line with the spatial locations of primary and secondary decidual zones described in the literature. To establish their claims, authors need to use known molecular markers for the different zones.
➔ We are showing immunostaining with an established primary decidual zone (PDZ) marker PTGS2 (COX-2) in the current Fig. 2 to demonstrate how the SHG signal does not originate from the PDZ. In addition to the E5.5 figure shown in the previous version of the manuscript, we have now also added time points E4.5 and E6.5 into this figure panel to better illustrate the point. We also show Masson's trichrome stainings to indicate that the SHG signal corresponds to the area with decidualized cells distinguished by their enlarged, multinuclear morphology. By this excluding method, we can state that the SHG signal is not stemming from the PDZ but rather from the more outer decidua, which usually is referred to as the secondary decidual zone (SDZ). However, definitions of the PDZ and SDZ are somewhat variable in the literature in terms of the embryonic days and corresponding areas covered, and on top of that, most schematic drawings of the different decidualization zones are vague at best (see, e.g., Paria et al. 2001 PNAS 98(3):1047-52; Yuan et al. 2019 Nat Commun. 10(1):5425; Favaro et al. 2014, The Guide to Investigation of Mouse Pregnancy, p. 125-142; Mori et al. 2016 Semin Immunopathol. 38(6):635-649; Maurya et al. 2021 Front Cell Dev Biol 9:640907; and Panja & Paria 2021 Adv Anat Embryol Cell Biol. 234: 205-221 to outline this discrepancy). Most sources seem to define SDZ establishment between E5 and E6 and its full peak at E8.

Regarding SDZ molecular markers, for instance, the (putative) SDZ defined by *Bmp2* in situ hybridization data exhibits gene expression pattern only partially overlapping with another accepted non-PDZ decidualization marker *Wnt4* (e.g., Paria et al. 2001 PNAS 98(3):1047-52; Yuan et al. 2019 Nat Commun. 10(1):5425). It is also critical to note that all data on *Bmp2* and *Wnt4* in decidua originates, to the best of our knowledge, from mRNA and not from protein expression studies. SHG outlines fibrillar collagen proteins, generating a signal delay in comparison to the accepted view of *Bmp2* and *Wnt4* gene expression, which precede the protein production, and is likely one reason why the view is different. In the new Fig. 1 we now more carefully present the matching of SHG signal with fibrillar collagen using Picrosirius red staining. As decidual fibrillar collagen is known to be deposited by the decidual cells, along with our histologic analyses, we can say that decidual SHG signal is highly specifically stemming from the area having actively proliferating non-PDZ decidual cells. We envision that the 3DMOUSEneST method can be used in the future to address the discrepancies to establish intrinsic clues for precise SDZ definition.

The authors coin the term decidual nests. However, it has been long known that collagens and ECM accumulates around the newly formed decidua.
➔ We appreciate this note by the reviewer and were also conscious when introducing a new term not to rename already known structures. While it is known that decidual cells deposit increased amounts of ECM, including fibrillar collagens, it has not been shown before how the fibrillar collagen forms a highly distinct 3D structure that in fact has value in the analysis of decidualization efficacy. We have made sure to acknowledge that ECM and collagen accumulation around decidual cells as such is well known by citing numerous related studies whenever applicable in the text, and we have now added a sentence even better recognizing this in the Introduction chapter (lines 86-87). However, we aim to point out that decidual collagen deposition and its biological role are still somewhat poorly studied. We believe that the lack of methodologies enabling this was holding back the advancement and that the 3DMOUSEneST methodology will have its place in facilitating such studies by providing an innovative readout for decidualization efficacy.

Authors claim that this methodology will indicate implantation efficiency. Implantation occurs between E4.0-E4.5 and decidual nests by name are indicative of decidualization. So, the method can predict if decidualization is happening
➔ We thank the reviewer for pointing out the need to use more fitting terminology. We have now changed this to be 'decidualization efficacy' throughout the manuscript.

© 2024. Published by The Company of Biologists under the terms of the Creative Commons Attribution License (https://creativecommons.org/licenses/by/4.0/).

The authors claim that this methodology is non-destructive. However, since the tissue is fixed and cleared there method is comparable to other techniques where samples are fixed and cleared after immunolabeling.

➔ We have now rephrased the text to remove the word 'non-destructive', which rightfully could cause confusion. We have indicated with other word choices that as the embryo is not dissected from the uterus, the implantation site remains intact, enabling its repurposing.

The authors claim that using this methodology will reduce usage of mice. However, since this is not live imaging animals will have to be dissected at different time points to acquire data as with other technologies that use fixed tissue and reconstruct 3D structures.

➔ We thank the reviewer for pointing out that this topic was poorly expressed in the manuscript. We intended to highlight that implantation site analysis with 3DMOUSEneST enables not only SHG/THG analysis of the conceptus and decidua but also sample repurposing to various complementing histologic techniques (new Fig. S2; Susaki et al. 2014 Cell 157(3):726-39; Tainaka et al. 2014 Cell 159(4):911-24; Nojima et al. 2017 Sci Rep. 7(1):9269), while allowing to keep the number of mice needed the same (no need to collect more mice for each different analysis method). In other words, it enables more data to be collected from a single implantation site if that is desired. If only 3DMOUSEneST is performed for base-level implantation site characterization, the number of euthanized mice is obviously not different than with other ex vivo methods. We have now corrected the text accordingly to make this clearer (lines 363-365 and 408-410).

It is possible to use previously cleared tissue for histology but the quality of histology for samples that are pre-cleared is not shown. To support their claim that material can be reused, the authors should perform SHG analysis on 3D cleared tissue and then provide 2D histology images to back the claim that the material can be used for tissue sectioning once SHG is done.

➔ It has been demonstrated earlier by other studies that immunohistochemistry, H&E staining, and electron microscopy are possible after CUBIC-1 clearance (Tainaka et al., 2014 Cell. 159(4):911-24.; Susaki et al. 2014 Cell 157(3):726-39; Nojima et al. 2017 Sci Rep. 7(1):9269), which we cite on lines 246-248. SHG and THG imaging are also known to have low toxicity on tissues (see, e.g., Campagnola 2011, Anal. Chem. 83(9):3224-3231; Aghigh et al. 2023 Biophys Rev. 15(1):43-70); this information was now added to the Introduction chapter (lines 96-100). Nevertheless, to illustrate that sample repurposing is possible for the specimens processed and imaged by the 3DMOUSEneST method (involving higher harmonic microscopy scanning in addition to CUBIC-1 clearing), we have now prepared a new figure panel (SFig. 2B,D,D') demonstrating sample repurposing for immunostaining after tissue clearing and the SHG/THG 3D scans are completed in comparison to the non-cleared implantation site (SFig. 2C,C').

Figure 2 Authors claim that in artificial decidualization fibrils are distributed sporadically compared to natural decidualization where collagen fibers have a more orderly organization. There is no quantification to back this data up. How many mice and how many decidual sites were evaluated?

➔ By presenting the data on artificial decidualization we show that 3DMOUSEneST method is sensitive for analysis of different patterns of collagen distribution, suggesting further applicability of the methodology. Most importantly, we demonstrate that the collagen deposition pattern differs in cases of artificial and natural decidualization, suggesting a critical role of an actual embryo for specific decidual collagen accumulation properties worth further investigation. Meanwhile, quantification of individual collagen fibrils goes beyond the aims of this study, as our main focus is on large-scale decidual nest volume measurements. We thank the reviewer for pointing out that we had missed reporting some of the specimen number information. We have now added the exact specimen number information on all figure legends and also added details to the 'Statistics' chapter of the Materials and Methods section. For this experiment, 5 control implantation sites and 4 artificial decidualization samples were imaged.

© 2024. Published by The Company of Biologists under the terms of the Creative Commons Attribution License (https://creativecommons.org/licenses/by/4.0/).

Based on the methods the samples from natural matings and those for artificial decidualization were fixed in different fixatives. Why were the samples not processed the same way? The organization of collagen fibers could easily be different because the tissue was fixed in different fixatives for the two samples being compared.

➔ We appreciate this careful observation, which we had missed mentioning, by the reviewer. We have used both 4% PFA and 10% neutral buffered formalin as fixatives in this study and did not observe notable differences in SHG emission. Information on used fixatives has now been updated in the Materials and Methods section. The two fixatives are often interchangeably used in biomedical research. However, to address the raised concern that the directly compared specimens in the current Fig. 5 were fixed in different fixatives possibly leading to altered fibrillar collagen arrangement in case of artificial decidualization specimens, we now present scans where both the natural implantation site and artificially decidualized uterus were fixed in the same fixative (10% formalin, new Fig. 5). Note that despite the changed fixative, fibrillar collagen arrangement remains sporadic in the artificial decidualization specimen in comparison to the natural implantation site (Fig. 5).

In figure 6 not all genotypes are shown in panel K for all the time points and it is unclear why some genotypes were omitted.

➔ *Smad1/5* cKO mice were studied at E5.5 since after that time point the implantation site number plummets indicating decidualization failure, which made *Smad1/5* cKO implantation sites well suitable for our proof-of-concept studies (lines 259-262). *Smad2/3* cKO mice were studied at E6.5 as that is the time point when implantation sites have a normal gross-morphological appearance, but lethality starts to appear (lines 266-272). Computer simulation allowed to model decidual nest usability in decidual efficacy evaluation without the need to collect E4.5 and E6.5 implantation sites from *Smad1/5* cKO mice (discussed in lines 392-394), thus overcoming difficulties in obtaining E6.5 *Smad1/5* cKO implantation sites and aiding in reducing the usage of mice when establishing new cohorts of mice would require extensive amount of time and financial input.

Do collagen fibrils in smadKOs organize normally or are they sporadic as the authors claim for artificial decidualization?

➔ The collagen fibrils are organized orderly around decidual cells in the case of *Smad* cKO mice, which can be seen from SFig. 3C-E, and since we did not observe a difference, the data is not presented separately from that point of view. However, the extent of decidualization progression is reduced in *Smad* cKO mice, seen as the reduced size of decidual nest volumes (Fig. 9).

Authors claim that the entire process is faster than other 3D protocols. What is the timeline for the entire protocol from dissection to imaging? What is the resolution of their method compared to published 3D methods.

➔ 3D MOUSEneST protocol is faster than those 3D protocols that involve whole mount antibody labeling. Based on our own work (e.g., Chi et al. 2011 PLoS One 6(11): e27676; Prunskaite-Hyyryläinen, 2019, Methods Mol Biol. 1926:185-199) and the work of others (e.g., Alanentalo et al. 2007 Nat Methods 4(1):31-3; Arora et al. 2016 Development 143(24):4749-4754; Yamaguchi et al. 2021. iScience 24(4):102258), whole mount antibody staining takes approximately 5-14 days, which can be completely omitted when intrinsic SHG signal is recorded, even though the other steps of the protocol (tissue collection, fixation, clearing, imaging, and analysis) are rather comparable. We have made sure that this difference is clear and comes across e.g. on lines 309-319 in discussion. We have now also added a timeline of the 3DMOUSEneST protocol in the new Fig. 4B and in the new Supplementary Materials and Methods. The resolution depends highly on the objective in use and can thus be altered depending on the needs. Decidual nest volume measurement does not require high resolution, but for more detailed imaging a higher magnification objective could be used. In this study, we used 16× objective for decidual nest analyses as indicated in the Materials and Methods section. The selection of objective and pixel resolution (1024×1024 in this study but this is possible to alter as desired) is also noted in the new step-by-step supplementary protocol (Supplementary Materials and Methods).

© 2024. Published by The Company of Biologists under the terms of the Creative Commons Attribution License (https://creativecommons.org/licenses/by/4.0/).

A big section in the introduction is duplicated.
➔ We have now deleted the unintentionally duplicated part.

Line 51 'Presently, many studies on early implantation sites still rely on traditional, labor-intensive two-dimensional (2D) microtome sectioning of paraffin-embedded tissue yielding only a few tissue sections for future analysis (Arora et al., 2016; Pang et al., 2014).' Arora et al 2016 is a study that describes 3D imaging of the entire uterine horn with immunolabeling and tissue clearing. It is not a 2D study.
➔ We thank the reviewer for helping us to realize that this sentence and the presented references might cause confusion to readers. We have now refined the sentence to add clarity (lines 48-51): 'Many studies on early implantation sites still rely on traditional, labor-intensive two-dimensional (2D) microtome sectioning of paraffin-embedded tissue yielding only a few tissue sections for future analysis-limitations, which have also been noted by others (Arora et al. 2016).' Arora et al. is referred to since also they have noted how 2D microtome sectioning, the golden standard method for implantation site analysis, has obvious limitations considering time and data output. Later in the text we also acknowledge the key work by Arora et al. 2016 for 3D imaging of immunolabeled implantation sites (lines 65-67).

Resubmission

First decision letter

MS ID#: DEVELOP/2024/202938

MS TITLE: 3DMOUSEneST: a volumetric, label-free imaging method evaluating embryo-maternal interaction and decidualization efficacy

AUTHORS: Audrey Savolainen, Emmi Kapiainen, Veli-Pekka Ronkainen, Valerio Izzi, Martin M Matzuk, Diana Monsivais, and Renata Prunskaite-Hyyrylainen

I have now received all the referees reports on the above manuscript, and have reached a decision. The referees' comments are appended below, or you can access them online: please go to BenchPress and click on the 'Manuscripts with Decisions' queue in the Author Area.

The overall evaluation is positive and we would like to publish a revised manuscript in Development. Both reviewers recommended publication, but Reviewer 2 has some outstanding comments that I hope you might address in the final version of the manuscript. Please attend to all of their comments in your revised manuscript and detail them in your point-by-point response. If you do not agree with any of their criticisms or suggestions explain clearly why this is so. Your revised manuscript will not require further review rather I will look it over myself prior to acceptance.

Reviewer 1

Advance summary and potential significance to field

The authors addressed all my concerns. They removed all confusing data and revised the description of the results. I don't have further questions.

Comments for the author

I don't have more suggestions.

Reviewer 2

© 2024. Published by The Company of Biologists under the terms of the Creative Commons Attribution License (https://creativecommons.org/licenses/by/4.0/).

Advance summary and potential significance to field

Authors have developed a methodology to evaluate decidual efficacy during early mouse pregnancy using a label free method. Based on first round of review, the authors have removed experiments/figures that were not supporting their claims.
They have additionally performed new experiments in response to reviewer comments.

Comments for the author

Major comments:

While the authors have provided supporting data that their method evaluates decidualization efficiency there is no evidence of embryo-maternal interactions.

This term is misleading and should be removed from the title (and other areas) of the manuscript.

In the abstract authors claim that their method allows evaluation of early conceptus (embryo and extraembryonic tissues) growth. Again there is absolutely no evidence of evaluating extraembryonic tissues and this reference should be removed from the abstract.

Collagens are highly expressed by the different uterine cell types (including epithelium and stroma) both peri-implantation and during decidualization. Thus line 136 “decidualization initiates and endometrial cells only start to differentiate into collagen-depositing decidual cells,” is again misleading. As SHG signal coincides with decidual collagens perhaps there is a certain kind of collagen (fibrillar?) that is closely associated with the SHG signal allowing this method to evaluate decidual efficiency. What kind of collagens contribute to fibrillar collagen? This should be part of the discussion.

Further in line 136 using endometrial cells is confusing – do the authors mean uterine epithelial cells? Or stromal cell? Or some other cells?

Line 151 – Authors state in the results that “decidual SHG-signal does not colocalize with PDZ”, however in the figure legend for Figure 2 the authors state that “Overlay of the PTGS2 staining with the SHG signal shows that the SHG signal does not significantly overlap with the PDZ”. The authors should be careful in overinterpretation of data and should relay the results as observed. This reviewer agrees with the statement in the figure legend. The statement in the results should be fixed.

Figure 3 – Authors have added Massons trichrome staining. The images show only 2 colors and it is hard to appreciate the classic tricolor. It is unclear to this reviewer what color of the masons trichrome is staining collagen and should overlap the SHG signal.

Figure 5 – When evaluating artificial decidualization, did the authors ensure that the thickness of the uterus post decidualization is similar to that of a E5.5 embryo? Based on the authors results themselves the level of SHG signal and density of fibrillar collagen is a direct indicator of the decidualization efficiency. Thus SHG signal increases form E4.5 to E5.5 to E6.5. Artificial decidualization by definition is an artificial process and the extent of decidualization varies from experiment to experiment. Thus it is difficult to compare data between a controlled development of an embryo with the oil stimulus.

These data are very hard to interpret given that the extent of artificial decidualization cannot be controlled.

Since the THG signal is strong in the surrounding endometrial tissues (line 204) and the signal is very weak in the conceptus (line 205), this reviewer is not convinced that other than size any information regarding the conceptus can be derived. Thus this method will only allow evaluation of conceptus size (and not embryo-maternal interactions). A reference to interactions should be removed throughout the manuscript.

© 2024. Published by The Company of Biologists under the terms of the Creative Commons Attribution License (https://creativecommons.org/licenses/by/4.0/).

For Figure 7, data from 5 implantation sites is reported. How many mice were used?

For the metrics presented – conceptus length/conceptus width/decidual nest depth/width, do these metrics stay consistent from one pregnancy to another? Do these depend on the number of implantation sites in the uterine horn? Do they change with mouse background? If any of these are true then the absolute values presented in the manuscript are less meaningful. If the ratios/percentages stay the same despite the variables mentioned above then those would be useful indicators of pregnancy progression.

Figure 8 – For all embryos that were called as failing, supplemental figure S3 shows that the signal for the THG in the endometrium was much less. Thus again there is no way to distinguish if the phenotypes are arising due to the endometrium or the embryo. Further this methodology cannot be used for predictions of pregnancy. It can only catch phenotypes that already exist as smaller decidual nests or smaller embryo size. It cannot be used to predict pregnancy progression.

These statements need to be revised to accurately demonstrate the strengths and limitations of the method.

The reference to implantation crypt throughout should be switched to implantation chamber (Madhavan et. al, Development, 2022). Crypts have been referred to invaginations on day 3.5 of mouse pregnancy (for eg. Cha et al, Cell Reports 2014) as well as to implantation chambers on day 4.5 of mouse pregnancy (for eg. Yuan et al, Nature communications, 2018) causing confusion in the literature.
Using the term implantation chamber will help clarify the structure that is being recognized by this method.

First revision

Author response to reviewers' comments

Response letter to reviewers' comments to Savolainen **et al.*****, manuscript***

Reviewer 1 *Advance Summary and Potential Significance to Field*:
The authors addressed all my concerns. They removed all confusing data and revised the description of the results. I don't have further questions.

Reviewer 1 Comments for the Author:
I don't have more suggestions.

Reviewer 2 Advance Summary and Potential Significance to Field:

Authors have developed a methodology to evaluate decidual efficacy during early mouse pregnancy using a label free method. Based on first round of review, the authors have removed experiments/figures that were not supporting their claims. They have additionally performed new experiments in response to reviewer comments.

Reviewer 2 Comments for the Author:

While the authors have provided supporting data that their method evaluates decidualization efficiency there is no evidence of embryo-maternal interactions. This term is misleading and should be removed from the title (and other areas) of the manuscript.

While we appreciate this point-of-view, we strongly believe our data and the published literature support the use of term 'embryo-maternal interactions' when describing 3DMOUSEneST. The term 'embryo-maternal interaction' and its derivatives, such as 'embryo-uterine', 'conceptus-uterine', or 'feto-maternal' interaction, have numerous definitions in the literature. In addition to the

© 2024. Published by The Company of Biologists under the terms of the Creative Commons Attribution License (https://creativecommons.org/licenses/by/4.0/).

interactions on the most immediate interface of the embryo and the luminal epithelium, initiation of the decidualization reaction in endometrial stromal cells upon blastocyst attachment is one of the commonly agreed readouts of this process (see, e.g., Lim & Wang 2010, J Clin Invest 120(4):1004-15) and is well captured by the 3DMOUSEneST method. Although 3DMOUSEneST does not detect the interaction on a molecular level or with single-cell resolution, we present a method that shows both the conceptus and the corresponding uterine decidual reaction occurring in response to embryo implantation (Figs 4-9; Movies 1-3). While visualizing and quantifying decidualization, the 3DMOUSEneST method is simultaneously a readout of the maternal reaction to the embryo. The 3DMOUSEneST method depicts the conceptus and fibrillar collagen deposited by decidualized cells in the secondary decidualization zone (SDZ), which is not in direct contact with the embryo. This means that the signals to decidualize are transduced to these cells via embryo-maternal/uterine interactions across several cell layers. Notably, in mice, natural decidualization occurs only as a response to an attaching blastocyst, further supporting the decidualization as an outcome of embryo-uterine interaction. Embryo-maternal/uterine interactions at the implantation sites can thus be considered to extend from the embryo to the decidua and vice versa (Chen et al., 2023, eLife 12:e82970; Ochoa-Bernal & Fazleabas 2020, Int J Mol Sci 21(6):1973; Sharma et al. 2016, Am J Reprod Immunol 75(3):341-50). Failure to sustain uterine growth and differentiation after blastocyst attachment is known to result in spontaneous abortion. Furthermore, the active and pregnancy-supporting role of extracellular matrix remodeling in the stromal endometrium and not just on the direct embryo-maternal/uterine interface (Favaro et al. 2014, The Guide to Investigation of Mouse Pregnancy, p. 125-142; Moraes et al. 2018, Proc Natl Acad Sci U S A, 115(8):E1749-E1758) goes often overlooked, and we hope our studies on its part increase this awareness. Moreover, even in vitro modeling of early pregnancy events has been described as studying the embryo-maternal/uterine interactions, further showing the broad spectrum of the term applicability (Schoen & Chen 2018, Anim Reprod, vol.15, nSupplement 1, p.784-790; Butt et al. 2024, Reprod Fertil Dev, 36(2):81-92). Considering the reviewer's comment, the literature, and consultations with other scientists, we conclude that 'embryo-uterine interaction' is the most acceptable term in the scope of this manuscript, putting emphasis specifically on the uterus where the decidual reaction takes place. Given this and the aim to increase terminological precision, we have now replaced the term 'embryo-maternal' to 'embryo-uterine' interaction throughout the manuscript and better defined these interactions in the manuscript text (lines 341-345).

In the abstract authors claim that their method allows evaluation of early conceptus (embryo and extraembryonic tissues) growth. Again there is absolutely no evidence of evaluating extraembryonic tissues and this reference should be removed from the abstract.

It might be that some of our data on extraembryonic tissues was accidentally overlooked. As we show in Fig. 6, the Reichert's membrane, the outermost membrane encasing the cells of embryonic origin in early mouse pregnancy (Matsuo et al. 2022, Phil. Trans. R. Soc. B 377:20210257), is directly encapsulating the structure that emits less THG signal, demonstrating that this 'dark hollow' is the conceptus. We are using the term conceptus as it encompasses both the embryo proper and the extraembryonic tissues (see, e.g., Hyun et al. 2020, Stem Cell Reports, 14(2):169-174). We do, however, understand that one could interpret the 'embryo and extraembryonic tissues' in parentheses meaning that we can observe and in detail measure those embryonic structures separately. Therefore, we have now modified the abstract to better indicate that with the 3DMOUSEneST method the conceptus can be examined as a whole entity (lines 30-31).

Collagens are highly expressed by the different uterine cell types (including epithelium and stroma) both peri-implantation and during decidualization. Thus line 136 'decidualization initiates and endometrial cells only start to differentiate into collagen-depositing decidual cells,' is again misleading. As SHG signal coincides with decidual collagens perhaps there is a certain kind of collagen (fibrillar?) that is closely associated with the SHG signal allowing this method to evaluate decidual efficiency. What kind of collagens contribute to fibrillar collagen? This should be part of the discussion.

We have now rephrased the sentence to be unambiguous and scientifically more accurate:

© 2024. Published by The Company of Biologists under the terms of the Creative Commons Attribution License (https://creativecommons.org/licenses/by/4.0/).

'Following implantation, the stromal endometrial cells gradually differentiate into decidual cells which deposit enhanced levels of thick collagen fibrils (Favaro et al., 2014), well corresponding with the progressively increasing levels of both SHG signal and Picrosirius red staining we observed around the implantation chamber from the time of implantation at E4.5 to decidualization at E5.5 and E6.5 (Fig. 1A-I)' (lines 139-144). SHG is a well-established method for imaging several types of fibrillar collagens that intrinsically have a non-centrosymmetric structure (see, e.g., Chen et al. 2012, Nat Protoc. 7(4):654-69). The specific collagen types increasingly produced in the decidua when compared to interimplantation sites have been reported to be fibrillar collagens I, III, and V (Spiess et al. 2007, Connect Tissue Res 48, 99-108.; Spiess & Zorn 2007 Microsc. Res. Tech. 70:18-25; Teodoro et al. 2003, Connect Tissue Res 44, 96-103). We mention this and cite relevant papers in our Introduction chapter (lines 86-89; 93-98).

Further in line 136 using endometrial cells is confusing - do the authors mean uterine epithelial cells? Or stromal cell? Or some other cells?

We have now defined that stromal endometrial cells are in question and modified the whole sentence to be clearer: 'Following implantation, the stromal endometrial cells gradually differentiate into decidual cells which deposit enhanced levels of thick collagen fibrils (Favaro et al., 2014), well corresponding with the progressively increasing levels of both SHG signal and Picrosirius red staining we observed around the implantation chamber from the time of implantation at E4.5 to decidualization at E5.5 and E6.5 (Fig. 1A-I)' (lines 139-144).

Line 151 - Authors state in the results that 'decidual SHG-signal does not colocalize with PDZ', however in the figure legend for Figure 2 the authors state that 'Overlay of the PTGS2 staining with the SHG signal shows that the SHG signal does not significantly overlap with the PDZ'. The authors should be careful in overinterpretation of data and should relay the results as observed. This reviewer agrees with the statement in the figure legend. The statement in the results should be fixed.

We have now corrected the sentence in the Results text to better reflect the data: 'Immunostaining of prostaglandin-endoperoxide synthase 2 (PTGS2), a known PDZ marker, revealed that the decidual SHG signal does not considerably colocalize with the PDZ (Fig. 2)' (lines 154-155).

Figure 3 - Authors have added Massons trichrome staining. The images show only 2 colors and it is hard to appreciate the classic tricolor. It is unclear to this reviewer what color of the masons trichrome is staining collagen and should overlap the SHG signal.
In Fig. 1, we have used Picrosirius Red staining to explicitly validate the overlap of the SHG signal with fibrillar collagen, and hence that is not the main focus in Fig. 3. Instead, Masson's trichrome staining in Fig. 3 is primarily intended to demonstrate how far the secondary decidual zone, comprised of differentiated decidual cells, is reaching in comparison to the SHG signal. Masson's trichrome provided good morphological contrast for distinguishing decidual cells (dark purple, large) from non-decidualized endometrial cells (light purple, small). When zoomed far out, the blue staining indicative of collagen is not so prominent in the images. Attached is a figure panel (***Fig. A***) with close-ups of the same staining to show that our Masson's trichrome staining is also picking up the collagen as is typical. To clarify any potential confusion, we have now added further explanation of the staining outcome into the Fig. 3 figure legend: 'The enlarged decidual cells appear as dark purple at E5.5 (E) and E6.5 (F) in comparison to the adjacent, non-decidualized endometrial stromal cells in lighter purple. Collagen stained in blue is not prominent at this magnification.'

© 2024. Published by The Company of Biologists under the terms of the Creative Commons Attribution License (https://creativecommons.org/licenses/by/4.0/).

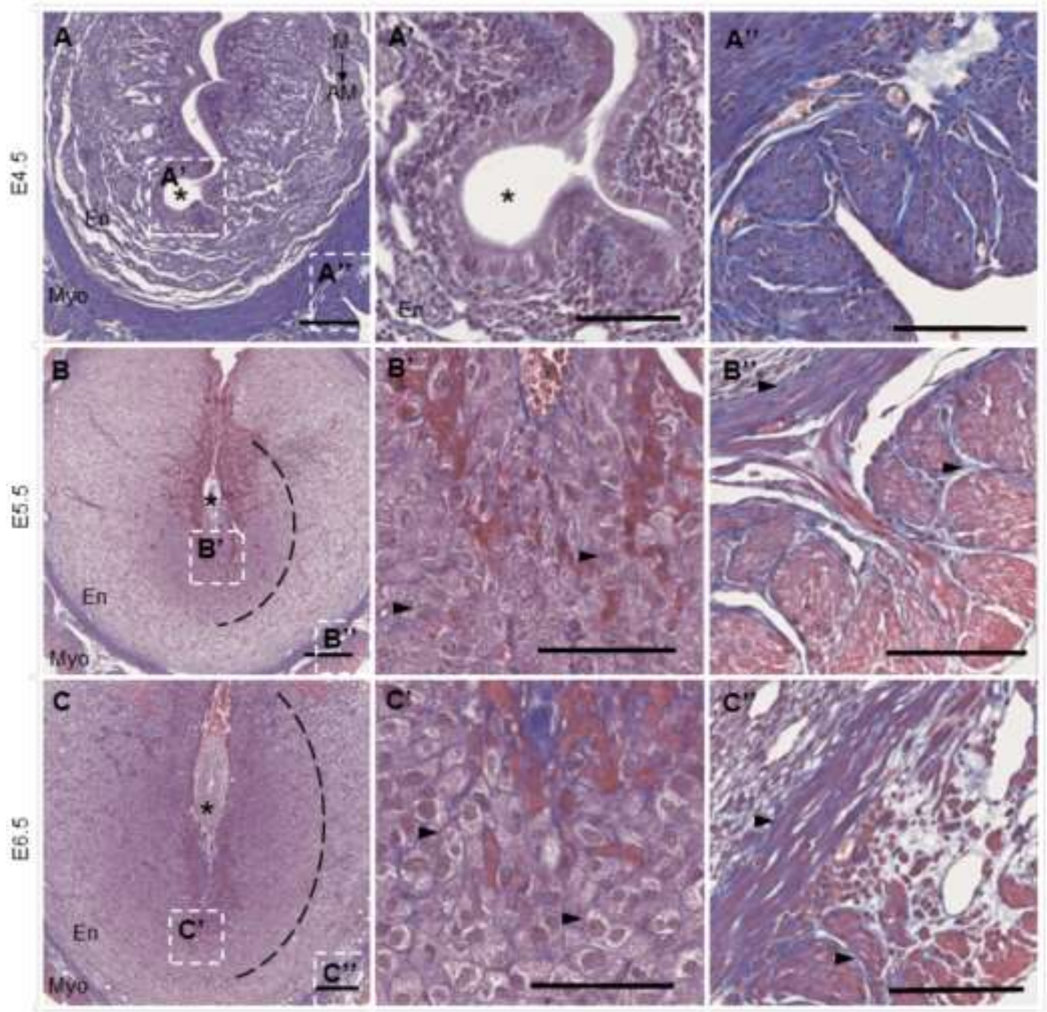

Fig. A. Clarification of Masson's Trichrome Staining in Fig. 3. (A-C) Original Masson's Trichrome images shown in Fig. 3 at E4.5-E6.5. (A'-C') Magnified areas showing E4.5 implantation chamber (A') and decidual cells (B'-C'), emphasizing blue collagen staining (arrows). (A''-C'') Magnified area showing the myometrium as a control for collagen staining (arrows). Asterisks mark the embryos and white dashed lines the magnified areas. E, embryonic day; En, endometrium; M, mesometrial; Myo, myometrium. Scale bars for A-C are 200 µm and for A'-C'' 100 µm.

Figure 5 - When evaluating artificial decidualization, did the authors ensure that the thickness of the uterus post decidualization is similar to that of a E5.5 embryo? Based on the authors results themselves the level of SHG signal and density of fibrillar collagen is a direct indicator of the decidualization efficiency. Thus SHG signal increases form E4.5 to E5.5 to E6.5. Artificial decidualization by definition is an artificial process and the extent of decidualization varies from experiment to experiment. Thus it is difficult to compare data between a controlled development of an embryo with the oil stimulus. These data are very hard to interpret given that the extent of artificial decidualization cannot be controlled.

The size of an artificially decidualized uterus sample and a natural implantation site are indeed difficult to match, nor is there a need to do so. In the case of natural implantation, decidualization is concentrically distributed around the embryo, forming a decidual nest. As artificial decidualization was induced by an injection of sesame oil (Tang et al. 2022, Endocrinology 163(5):bqac043), the artificial decidual stimulus extended throughout the uterine horn. Because of the differences in the decidualization area, we did not attempt to quantify artificial decidualization in the same manner as the decidual nest. This was neither our objective nor feasible as in artificially decidualized uteri the SHG signal proved to be too sparse to generate such a distinct, measurable structure as the decidual nest is. By showing data from artificial

© 2024. Published by The Company of Biologists under the terms of the Creative Commons Attribution License (https://creativecommons.org/licenses/by/4.0/).

decidualization, (i) we provide evidence that SHG imaging is sensitive enough to detect differences in fibrillar collagen deposition appearing both in the case of natural and artificial decidualization. The similarities and differences between the two are well documented at the molecular level (Wang et al. 2020, Genes 11(8):935) but not in the context of extracellular matrix. (ii) We show that the SHG signal is also emitted by artificially decidualized uteri suggesting that it can be used as an alternative to identify the decidualized area in studies relying on artificially decidualized uteri experiments. Currently the studies use antibodies (desmin) or histological stainings to differentiate and quantify decidualized vs. non-decidualized areas (Peterse et al. 2018, Reprod Sci, 25(11):1577-1588). We hope this reasoning is sufficient justification for presenting the applicability of SHG imaging in studies analyzing artificially decidualized uteri. We double-checked that in the text we discuss only the observed qualitative but not quantitative SHG signal differences between natural and artificial decidualization.

Since the THG signal is strong in the surrounding endometrial tissues (line 204) and the signal is very weak in the conceptus (line 205), this reviewer is not convinced that other than size any information regarding the conceptus can be derived. Thus this method will only allow evaluation of conceptus size (and not embryo-maternal interactions). A reference to interactions should be removed throughout the manuscript.

Please see the more detailed answer to the first comment on embryo-maternal interactions above. Both the conceptus and the decidual collagen accumulation can be visualized and measured simultaneously. Alterations of the conceptus size in conjunction with the decidual response reflect the normal growth of the embryo. We have now defined this in more detail in the text (lines 341-345) and replaced the more general term ‘embryo-maternal interactions’ with more accurate ‘embryo-uterine interactions’ throughout the manuscript.

For Figure 7, data from 5 implantation sites is reported. How many mice were used? For the metrics presented - conceptus length/conceptus width/decidual nest depth/width, do these metrics stay consistent from one pregnancy to another? Do these depend on the number of implantation sites in the uterine horn? Do they change with mouse background? If any of these are true then the absolute values presented in the manuscript are less meaningful. If the ratios/percentages stay the same despite the variables mentioned above then those would be useful indicators of pregnancy progression.

The implantation sites for this experiment were collected from three different female mice at E4.5 and E5.5 and from two female mice at E6.5. The number of dams used in each experiment in this manuscript to obtain implantation sites was at least two and the number of implantation sites at least three. We report relevant information in the Methods section (lines 660-663) and in the figure legends. Since the dam is always euthanized when implantation sites are collected, we cannot follow the consistency of the measurements across several pregnancies of one dam. There is natural variability in conceptus and implantation site sizes, which is visible in decidual nest size as well and is reflected in our studies as ± standard deviations. We have not in detail examined the effect of the number of implantation sites in the uterine horn on these metrics. This would be an interesting topic for future studies. We have unpublished conceptus/decidual nest metrics data from E5.5 C57BL/6J and E5.5 C57BL/6J/129S5SvEvBrd mouse strains and we did not observe any significant differences between them, as is shown in the figure (***Fig. B***) below. For this reason, we did not specifically report this data. Given this, when herein reported mouse strains are studied, scientists can use these values as reference points. However, this does not exclude the fact that differences might exist in other strains that are worth analyzing in the future. We envision generation of decidual nest size data for the most commonly used mouse strains to serve as a reference point. Furthermore, in most studies, experimental and control animal groups are from the same strain, and scientists applying the method in following research will be able to obtain internal control values and do comparisons based on those. The data we present in Fig. 7 emphasizes how versatile information the method offers and describes how the previously uncharacterized decidual nest develops in tandem with the conceptus.

NOTE: We have removed unpublished data that had been provided for the referees in confidence.

© 2024. Published by The Company of Biologists under the terms of the Creative Commons Attribution License (https://creativecommons.org/licenses/by/4.0/).

Fig. B. Ratios of conceptus and decidual nest measurements in two different mouse strains, C57BL/6J and C57BL/6J/129S5/SvEvBrd, at E5.5.

Figure 8 - For all embryos that were called as failing, supplemental figure S3 shows that the signal for the THG in the endometrium was much less. Thus again there is no way to distinguish if the phenotypes are arising due to the endometrium or the embryo.

We use endometrial THG signal as a guide to detect the conceptus. Its intensity can be variable across different samples, but it is consistently markedly lower in the conceptus and can thus be used to define its boundaries. We have validated this in Fig. 6 using laminin staining to detect the embryo-encapsulating Reichert's membrane together with THG analysis. In Fig. 6D, we showcase the THG intensity difference between the conceptus and the surrounding tissues; however, when using 3DMOUSEneST, the exact THG intensity is not relevant for measuring conceptus dimensions from digital slice images. THG signal stems from various water-lipid and water-protein interfaces in the samples and is affected by, e.g., sample size and depth, which can explain the biological variability in its intensity even in seemingly comparable tissues (Weigelin et al. 2016 J Cell Sci 129(2):245-55). Due to this variability, the threshold for epi THG is not standardized but it was set to illustrate the conceptus border. We have now added better explanation of THG thresholding and signal processing into the Methods section (lines 609-618). In the experimental setup in Fig. S3, we used *Smad* cKO female mice, which are already previously phenotyped to have defects in their decidua and early pregnancy maintenance. Noteworthily, Fig. S3 displays samples from two different age points, which may affect the physical properties of the uterus and thus the THG signal (see also THG signal in Fig. 4 for several age points). We have now also added a new Movie (Movie 1) that displays the z-stack image collection through the implantation sites, further clarifying to the readers how the conceptus (THG) and decidual nest (SHG) appear across the samples before 3D reconstruction.

Further this methodology cannot be used for predictions of pregnancy. It can only catch phenotypes that already exist as smaller decidual nests or smaller embryo size. It cannot be used to predict pregnancy progression. These statements need to be revised to accurately demonstrate the strengths and limitations of the method.

We appreciate the reviewer for bringing this up. Indeed, this method is static as it captures a fixed moment in embryo-uterine interaction and does not allow in vivo follow-up of the same implantation site to get true data on the pregnancy outcome, and this is an obvious limitation. Nevertheless, this method allowed us to identify measurable parameters (decidual nest and conceptus size) that correlate with pregnancy progression as demonstrated in the examples of *Smad* KO mice with known fertility issues. We believe that as the method usage expands and a greater variety of phenotypes is analyzed, we will be able to find out even more parameters and patterns allowing their correlation with pregnancy progression and even association with remaining pregnancy survival time. This is an exciting possibility but remains to be studied and seen. In the current manuscript, when talking about the data derived from the logistic regression (LR) analysis model we have made sure not to overstate its power, and, as examples, refer only that it predicts 'the likelihood of early pregnancy progression to the next developmental stage' or that 'decidual nest volumes associate with the potential to progress to the next pregnancy day or fail' (lines 286-287; 402-403). Furthermore, we added text discussing the prognostic limitations of the LR method in the Discussion chapter: 'It is worth noting that the LR prediction has its limitations as it will provide only a theoretical pregnancy progression likelihood, for which *in vivo* validation is not possible as 3DMOUSEneST analyses are conducted *ex vivo* using already fixed specimens' (lines 403-405).

The reference to implantation crypt throughout should be switched to implantation chamber (Madhavan et. al, Development, 2022). Crypts have been referred to invaginations on day 3.5 of mouse pregnancy (for eg. Cha et al, Cell Reports, 2014) as well as to implantation chambers on day 4.5 of mouse pregnancy (for eg. Yuan et al, Nature communications, 2018) causing

© 2024. Published by The Company of Biologists under the terms of the Creative Commons Attribution License (https://creativecommons.org/licenses/by/4.0/).

confusion in the literature. Using the term implantation chamber will help clarify the structure that is being recognized by this method.

We thank the reviewer for this insightful comment. We have now changed the term to be implantation chamber throughout the manuscript and added a reference to the recent hallmark paper Madhavan et al. upon explanation of the implantation chamber in the Introduction chapter (lines 76-78).

Second decision letter

MS ID#: DEVELOP/2024/202938

MS TITLE: 3DMOUSEneST: a volumetric, label-free imaging method evaluating embryo-maternal interaction and decidualization efficacy

AUTHORS: Audrey Savolainen, Emmi Kapiainen, Veli-Pekka Ronkainen, Valerio Izzi, Martin M Matzuk, Diana Monsivais, and Renata Prunskaite-Hyyrylainen
ARTICLE TYPE: Techniques and Resources Article

I am happy to tell you that your manuscript has been accepted for publication in Development, pending our standard publication integrity checks.

© 2024. Published by The Company of Biologists under the terms of the Creative Commons Attribution License (https://creativecommons.org/licenses/by/4.0/).
